# Erasable and Field Programmable DNA Circuits Based on Configurable Logic Blocks

**DOI:** 10.1002/advs.202400011

**Published:** 2024-05-02

**Authors:** Yizhou Liu, Yuxuan Zhai, Hao Hu, Yuheng Liao, Huan Liu, Xiao Liu, Jiachen He, Limei Wang, Hongxun Wang, Longjie Li, Xiaoyu Zhou, Xianjin Xiao

**Affiliations:** ^1^ School of Life Science and Technology Wuhan Polytechnic University Wuhan 430023 China; ^2^ Institute of Reproductive Health Tongji Medical College Huazhong University of Science and Technology Wuhan 430030 China; ^3^ Department of Precision Diagnostic and Therapeutic Technology City University of Hong Kong Shenzhen Futian Research Institute Shenzhen Guangdong 518000 China; ^4^ Department of Laboratory Medicine Tongji Hospital Tongji Medical College Huazhong University of Science and Technology Wuhan 430030 China

**Keywords:** clip‐mediated strand displacement reaction, configurable logic block, DNA logic circuits, DNA nanotechnology, field programming

## Abstract

DNA is commonly employed as a substrate for the building of artificial logic networks due to its excellent biocompatibility and programmability. Till now, DNA logic circuits are rapidly evolving to accomplish advanced operations. Nonetheless, nowadays, most DNA circuits remain to be disposable and lack of field programmability and thereby limits their practicability. Herein, inspired by the Configurable Logic Block (CLB), the CLB‐based erasable field‐programmable DNA circuit that uses clip strands as its operation‐controlling signals is presented. It enables users to realize diverse functions with limited hardware. CLB‐based basic logic gates (OR and AND) are first constructed and demonstrated their erasability and field programmability. Furthermore, by adding the appropriate operation‐controlling strands, multiple rounds of programming are achieved among five different logic operations on a two‐layer circuit. Subsequently, a circuit is successfully built to implement two fundamental binary calculators: half‐adder and half‐subtractor, proving that the design can imitate silicon‐based binary circuits. Finally, a comprehensive CLB‐based circuit is built that enables multiple rounds of switch among seven different logic operations including half‐adding and half‐subtracting. Overall, the CLB‐based erasable field‐programmable circuit immensely enhances their practicability. It is believed that design can be widely used in DNA logic networks due to its efficiency and convenience.

## Graphical abstract

Inspired by Configurable Logic Block (CLB), the CLB‐based erasable field‐programmable DNA circuit, utilizing clip strands as operation‐controlling signals, enables diverse functions with limited hardware. The system is demonstrated to allow multiple rounds of field‐programming of seven logic operations, including half‐adder and half‐subtractor. The CLB‐based circuit is the first truly field programmable DNA circuit, offering efficiency and convenience for various applications.

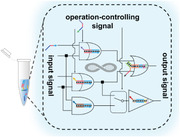

## 1 Introduction

As one of the most important branches in the field of bioengineering, artificial logic networks are committed to using molecules to simulate the logic operations of silicon‐based digital circuits with the ultimate goal of constructing submicroscopic computers.^[^ ^1^, ^2^, ^3^, ^4^, ^5^ ^]^ While DNA, apart from serving as a carrier of genetic information for disease diagnosis^[^ ^6^, ^7^, ^8^, ^9^ ^]^ and prognosis monitoring,^[^ ^10^, ^11^ ^]^ has become an excellent raw material for building biological computers due to its excellent biocompatibility.^[^ ^12^, ^13^, ^14^, ^15^ ^]^ At the same time, the Watson–Crick base pairing principle gives DNA a high degree of programmability. Therefore, researchers may precisely tune the thermodynamic and kinetic parameters of the DNA hybridization processes.^[^ ^16^, ^17^, ^18^, ^19^, ^20^, ^21^, ^22^, ^23^ ^]^ Although DNA circuits currently lag behind traditional silicon‐based circuits in terms of speed and scalability, their remarkable biocompatibility, nanoscale dimensions, and relatively low cost confer significant advantages for in vivo applications. Therefore, they still hold substantial untapped potential for further development (Discussion S2, Supporting Information). Owing to the advantages listed above, DNA is frequently used as a substrate for the construction of logic circuits.^[^ ^24^, ^25^, ^26^, ^27^, ^28^ ^]^ When building a DNA‐based logic circuit, researchers frequently take the following procedures: First of all, the researchers make the logical truth table according to their own needs and then design the digital circuit diagram. After that, by ingeniously designing the sequence of DNA strands, the researchers can transform the digital circuit diagram into a DNA reaction network,^[^ ^29^, ^30^ ^]^ which is often based on toehold‐mediated strand displacement (TMSD),^[^ ^19^, ^30^, ^31^, ^32^, ^33^, ^34^, ^35^ ^]^ or enzyme‐catalyzed reactions.^[^ ^36^, ^37^, ^38^ ^]^

Until now, several kinds of DNA reaction networks with their advantages have been proposed. The seesaw gate,^[^ ^4^, ^33^ ^]^which was proposed by Qian and Winfree, rendered the basic OR and AND gate structurally homogeneous.^[^ ^39^ ^]^ As a result, rather than using a totally different reaction principle, researchers only need to adjust the concentrations of the threshold gate to enable a seesaw gate circuit to perform different logic operations. This greatly simplifies the design complexity of DNA circuits. Later on, the polymerase‐based single‐strand gate provided a new idea, that is, to complete the strand displacement by an enzyme‐catalyzed reaction.^[^ ^38^ ^]^ Benefiting from the high efficiency of enzyme catalysis, the speed of DNA calculation has been greatly improved so that even the 4‐bit square root operation can be completed within 1 h. Overall, current DNA reaction networks, benefiting from their robustness and scalability, have been able to perform a variety of complex logic operations. However, until now, the DNA circuits have been disposable and lack field programmability. The researchers have to rebuild the entire circuit if the desired logic operation changes, which greatly restricts the practicability of DNA circuits. To solve the above bottleneck problem, researchers managed to erase and reuse DNA circuits by means of enzymatic, nonenzymatic, or photonic regulation.^[^ ^40^, ^41^, ^42^, ^43^, ^44^, ^45^, ^46^ ^]^ However, these circuits could only be reused with a single logic and did not achieve field programming of various logic functions. Pei's molecular automaton,^[^ ^47^ ^]^ though realizing field programming, can only be used once, which makes its field programming unpractical. Moreover, the scalability of this system was limited because it was unable to output DNA strands for cascaded reactions. To sum up, DNA circuits have been developing rapidly so that they can perform advanced logic functions. However, there is an urgent need to establish an erasable and field‐programmable DNA circuit to enhance its practicability.

**Figure 1:**
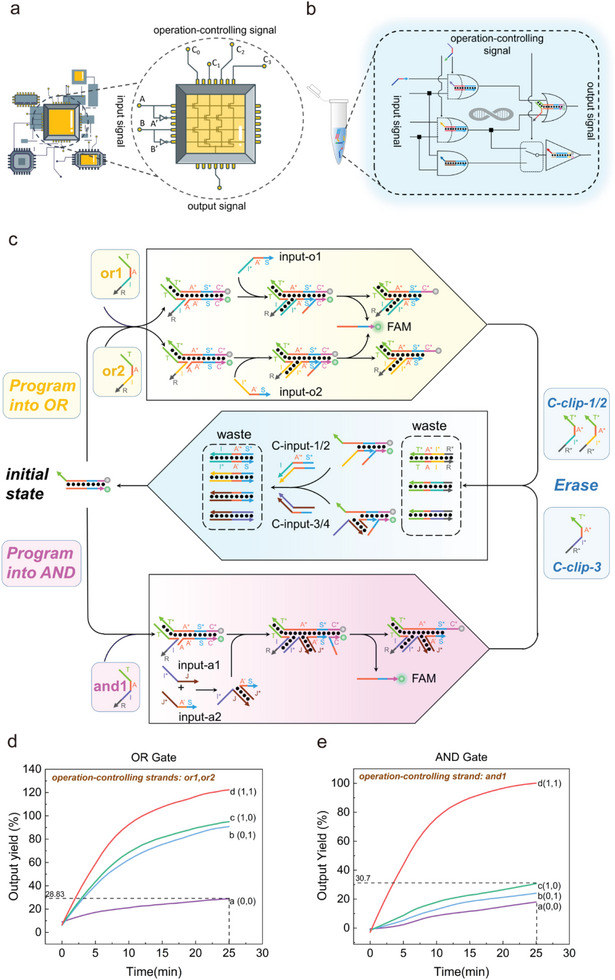
a) Schematic illustration of the Configurable Logic Block (CLB) in digital circuits. b) Schematic illustration of CLB‐based erasable field‐programmable DNA circuits. The clip strands were used as the operation‐controlling signals so that adding different clips could result in different logic operations. Also, the clip strands enabled the circuit to be erased by adding their complementary strands. c) Schematic illustration of CLB‐based erasable field‐programmable OR and AND gate. Adding different clips (operation‐controlling strands) would lead to corresponding logic operations, and the initial state could be restored by adding C‐clips and C‐inputs. X^*^ and X were designed to be complementary, where “X” stands for different domain names. d) Experimental verification of OR gate. e) Experimental verification of AND gate. Reaction setup: 100 nm (5 pmol) FAM:BHQ,120 nm (6 pmol) clips, 240 nm inputs (12 pmol) were added sequentially to form a system with final volume of 50 µL. All experiments depicted in the figure were replicated at least twice, with consistent trends observed.

To achieve the aforementioned goal, digital circuits give us a good inspiration: the Configurable Logic Block (CLB). Essentially, it is a function module, based on which circuits have the potential to perform a variety of logic operations, and the actual logic of the circuit is determined by a set of operation‐controlling signals.^[^ ^48^ ^]^ The corresponding relationship between the operation‐controlling signals and the logic operation is presented in the form of a table. Thus, field programming will be so simple that researchers will only need to check the table to find the matching operation‐controlling signals of the target logic operation and add them to the CLB. As an example, **Figure** **1**a shows a dual‐rail two‐input CLB. A, A', B, and B' are the input signals, while O is the output signal, and (C0,C1,C2,C3) is a 4‐bit operation‐controlling signal. Only when the value of the signal is 1, its corresponding logical relationship can be transmitted to the output. As shown in Table S1 (Supporting Information), the different combinations of operation‐controlling signals and their corresponding logical functions are listed. If a researcher wants to obtain an XOR circuit, he will not have to consider the arrangement and routing of the basic logic gate but will instead directly look up the table and input (0,0,1,1) to the CLB at the operation‐controlling end. With the advantage that the circuits can be erased and reprogrammed multiple times, users may realize various logic functions with limited hardware resources. Conclusively, constructing CLB‐based DNA circuits with reference to digital circuits would be an important development direction in the field of molecular computing.

Herein, we utilized the allosteric‐clip‐toehold mediated strand displacement reaction, which was previously proposed by our group,^[^ ^40^ ^]^ to realize the CLB‐based erasable and field‐programmable DNA circuit. As shown in Figure 1b, by regarding the clip strands as the operation‐controlling signals, we realized the precise correspondence between the logic functions and the controlling signals. In cascaded DNA circuits, we could set up multiple clip addition sites, just like the positions C0∼C3 in Figure 1a. At each site, only clip strands could provide the toehold for the invading strand to complete the strand replacement reaction. Consequently, adding clips with different toeholds may result in different reactions, i.e., field program the whole circuit as the researchers’ need. Considering the convenience, we listed the relationship between the clips and the logic operations in a table for the researchers’ query. Furthermore, our previous experiments have proved that complementary strands of clips and inputs can erase the circuit to its initial state. Taking advantage of this, we could add another set of clips according to the table after the erasing to reprogram the circuit to another logic function. To sum up, researchers can field program the CLB‐based erasable DNA circuits multiple times to obtain different logic functions according to their needs.

## 2 Result and Discussion

### 2.1 Construction of CLB‐Based Primary Logic Gates

For logic gate circuits, AND and OR gates are two fundamental logic gates because together they are sufficient to build universal Boolean logic.^[^ ^33^ ^]^ First of all, we explored how to construct such basic logic gates in a CLB mode. We used the previously reported allosteric clip‐mediated strand displacement reaction as the basic principle to realize those two logic gates. As shown in Figure S1a (Supporting Information), an allosteric clip consists of the following domains from 5′ to 3′: domain T with 14‐nt for binding with gate:output, domain A with 7‐nt for the allosteric reaction, domain I with 14‐nt for binding with input, and finally domain R with 8‐nt for the reset reaction. The clip was first hybridized with gate:output through domain T. Then the domain A and gate:output would undergo an allosteric reaction, a strand displacement reaction that would open the proximal end of the double strand DNA (dsDNA) and thereby assist the input strand to invade the dsDNA by taking domain I as a toehold. Figure S1b,c (Supporting Information) illustrated that when the concentration of FAM:BHQ duplex was 100 nm, the addition of a 120 nm clip and 240 nm input to the reporter would be enough to obtain an adequate output.

Here, we chose the allosteric clip over the traditional clip to speed up the reaction while reducing crosstalk. Figure S2a (Supporting Information) showed the principle of a  two‐layer circuit realized by clips and allosteric clips, respectively. The principle based on the traditional clip may cause crosstalk between input and output, thereby interfering with the desired reactions. Moreover, according to the fluorescent intensity shown in Figure S2b (Supporting Information), the allosteric clip apparently increased the conversion ratio of the reaction significantly.

After determining the reaction principle, we then tried to construct the two basic logic gates: OR gate and AND gate. As shown in Figure 1c, the OR gate was made up of two clips with different domain I and a gate:output duplex. Each clip was able to hybridize with the gate:output, and its corresponding input could initiate the clip‐mediated strand displacement, executing the function of an OR gate. As for the AND gate, it consisted of a gate:output duplex, a clip strand (and1), and two input strands (input‐a1 & input‐a2). The first step was the combination of clip and gate:output as well, then part of the input‐a1 would hybridize with domain I. While input‐a2 could hybridize with input‐a1 through a complementary section of 14‐nt and provide the strand‐displacement section. Consequently, together with input‐a1, input‐a2 could invade gate:output and displace the output strand. In other scenarios, when either input existed alone, the conversion ratio and rate of the strand displacement reaction would be extremely low due to the lack of a toehold or strand displacement section. The experimental results in Figure 1d,e demonstrated the function of the proposed logic gate in dealing with different inputs. For the basic one‐layer circuit, allosteric clip‐based strand displacement reactions possessed a considerable reaction rate, so that either logic could be completed within half an hour. At the same time, its good signal‐to‐noise ratio made it possible to implement subsequent reprogramming and cascaded circuits. Furthermore, the reaction speeds of the two logic gates were essentially equal, despite the fact that the AND gate required more complexity than the OR gate since the inputs needed to be complementary. This ensured that in a cascaded circuit, the output time of the logic gates at the same layer was basically identical. In a word, using allosteric clips, we demonstrated the feasibility of our proposed basic CLB‐based logic gates: the AND gate and the OR gate.

We would also like to discuss the advantages of our proposed logic gates. The core innovation of the CLB‐based circuit was the newly proposed AND gate sharing the same gate:output strands with the OR gate. Therefore, a gate:output duplex possesses the potential for both OR and AND operations. Based on this, we have introduced the concept of operation‐controlling signals for the first time. By implementing such signals in the system, the specific logic executed by gate:output is determined, enabling true field programming. Finally, we leverage the clip‐based strand displacement reactions previously proposed by us to confer resettability upon our system. In contrast, while some previous studies share similarities with ours in terms of reusing circuits by introducing DNA strands, their principles did not endow a single gate with the potential for multiple logic operations, nor did they incorporate operation‐controlling strands to program the logic executed by the gate. Consequently, these studies could only repeat the same logic multiple times but lacked the capability for logic switching.

### 2.2 The Erasability and Field Programmability of CLB‐Based Circuits

**Figure 2:**
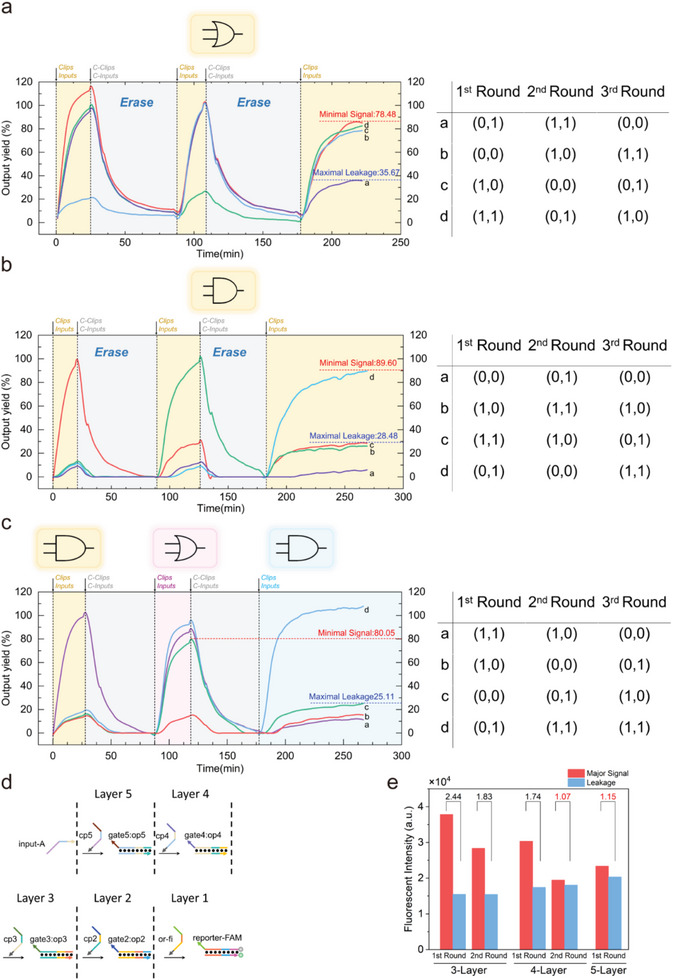
Experimental verification of the field programmability of the CLB‐based OR and AND gate. a) The fluorescent curves of the OR gate reusing for 3 times. b) The fluorescent curves of the AND gate reusing for 3 times. c) The fluorescent curves of the logic gate switched in the order of AND‐OR‐AND. Reactions setup: 100 nm (5 pmol) FAM:BHQ, 120 nm (6 pmol) operation‐controlling strands, 240 nm (12 pmol) inputs were added sequentially to form a system with a final volume of 50 µL for the first‐round using; 120 nm (6 pmol) C‐clips, 240 nm (12 pmol) C‐inputs for the first‐round erasing. The concentration of inputs/clips and C‐input/clips would increase 10 nm each round. d) The schematic illustration of the multilayer Translator system. e) Experiment Results of first (and second) round running for multilayer translator circuits. Reaction setup: 100 nm (5 pmol) FAM:BHQ, 120 nm (6 pmol) or‐fi was added. Afterward, the gate:output and clip, or input, of the upper layer circuit were added, with a concentration twice that of the lower layer. The final volume for the first‐time use was 50 µL. For erasability, the complementary strands (c‐clips and c‐inputs) were added with equal quantity to the clip and input strands in the forward reaction. For each round's reusing, the clip and input strands were added with a concentration increase of 10 nm compared to the previous round. All experiments depicted in the figure were replicated at least twice, with consistent trends observed.

We then would like to verify the erasability and field programmability of the CLB‐based circuit. According to our previous research, the initial state of the circuit could be returned by adding the complementary strands of clip (C‐clip) and input (C‐input). The detailed mechanism is illustrated in Figure 1c. C‐clip, taking Domain R as a toehold, reacted with the clip strand to form a waste duplex Clip: C‐clip. The output strand then dissociated the input via TMSD. At the same time, the hybridization of C‐input and input also promoted the separation of input and gate. In this way, any kind of logic can be erased to its “initial state”, where only gate:output and wasted dsDNA exist. Gate:output can be further used, but wasted Clip: C‐clip and Input: C‐input are blunt‐ended and inert. So far, the reaction system has been fully erased to its initial state. Then, the circuit is ready for the next round of field programming. The results of reusing and field programming the circuit are shown in **Figure** **2**a–c, respectively. First, 100‐nm FAM:BHQ duplex was added. Then, 120‐nm operation‐controlling strand (or1 & or2 for OR gate, and1 for AND gate) and 240 nm their corresponding input (input‐o1 & input‐o2 for OR gate, input‐a1 & input‐a2 for AND gate) were added. The reaction was considered sufficient when the fluorescence curve reached a plateau. After that, C‐clip (c‐clip‐1 & c‐clip‐2 for OR gate, c‐clip‐3 for AND gate) and C‐input (c‐input‐1 & c‐input‐2 for OR gate, c‐input‐3 & c‐input‐4 for AND gate) with the same concentration as clip/input were added to erase the circuit's logic functions to its initial state. Hence, a new round of operation‐controlling strands and input strands could be added, which could either be different or the same as the last round. In order to demonstrate the versatility of this field programmability, we enumerated all possible inputs for each logic: (0,0) (0,1) (1,0), and (1,1). The output results of each round were in line with the predicted outcomes. As the clip and C‐clip were sequentially added to the reaction system, the fluorescence signals rose and fell accordingly, and the amplitude of the fluorescence signal did not significantly attenuate. The fluorescent signal of each round was close to 100% yield, accompanied by a clear distinction between the major signal and the leakage. In terms of reaction rate, all of the forward reactions were completed within 30 min. Overall, the above data proved that a CLB‐based circuit could be erased and field programmed between AND and OR logic gates.

In order to test the size limit of the CLB circuit, we designed a multilayer Translator system. As shown in Figure 2d, this system is formed by connecting multiple translators in series. We used the CLB circuit to perform 3‐, 4‐, and 5‐layer translator systems, respectively. As shown in Figure 2e, as the number of layers increased, the major signal gradually declined and the leakage increased. As a result, the CLB circuit failed to reset when running a 4‐layer Translator system, and totally broke down when running a 5‐layer Translator system, because it cannot clearly distinguish the major signal form leakage in the above cases.

It's important to note that we increased the clip and input concentrations in each round by 10 nm compared to the previous round in order to lessen the impact of waste strands on the reaction rate and extent. We noted that during the reprogramming process, the volume increase caused by sample addition may dilute the fluorescence intensity. So, we corrected the fluorescence signals according to the volume variation and then calculated the output percentage of each forward reaction. The formula was as follows: $S_{A}=\frac{S_{0}\times V_{A}\left(\mu L\right)}{50(\mu L)}\;$, where *S*_0_ stood for the raw fluorescence signal value, *V_A_* for the volume of the sample at the time of measurement, and *S_A_* for the corrected fluorescence signal.

### 2.3 A Two‐Layer “X‐AND” Cascaded Circuit Based on CLB Logic Gates to Realize Multiple Logic Operations

**Figure 3:**
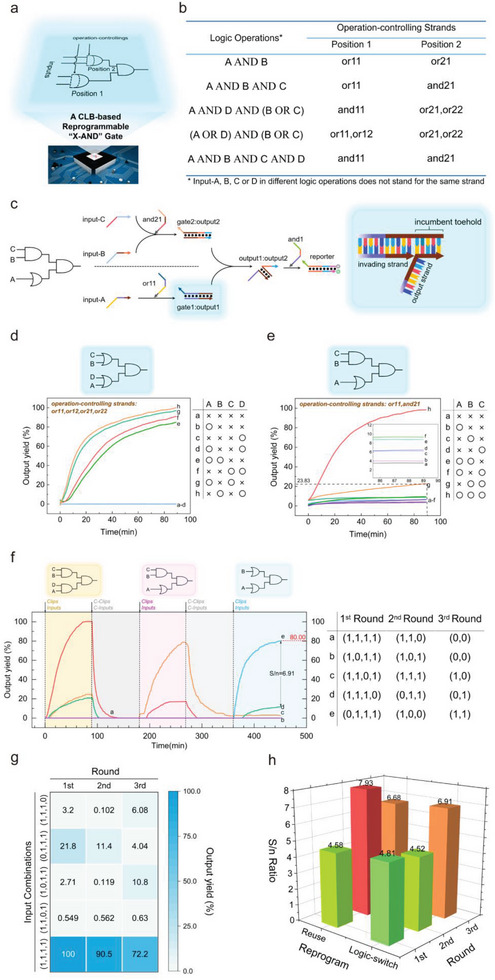
a,b) Schematic illustration and logic operation table of the CLB‐based “X‐AND” gate. There are two positions for users to add operation‐controlling strands to realize five different logic operations. c) Digital circuit diagram of “A AND B AND C” and the schematic illustration of its corresponding CLB‐based DNA circuit. The pattern inside the blue box shows the location of the “incumbent toehold”. d,e) The fluorescent curves of “(A OR D) AND (B OR C)”and “A AND B AND C”. Reactions setup: 100 nm (5 pmol) FAM:BHQ, 240 nm (12 pmol) gate2:output2 and gate1:output1, 120 nm (6 pmol) and1, 240 nm (12 pmol) operation controlling strands and inputs were added sequentially to form a system with final volume of 50 µL. f) The fluorescent curves of “X‐AND” gate switched in the order of (A AND D AND B AND C)‐(A AND B AND C)‐(A AND B). g) The heat map reflects the output yield of “A AND B AND C AND D” in a 3‐round reusing. h) The comparison of signal‐to‐noise ratio between logic‐switching and reusing “A AND B AND C AND D”. Reactions setup: 100 nm (5 pmol) FAM:BHQ, 240 nm (12 pmol) gate2:output2 and gate1:output1, 120 nm (6 pmol) and1, 240 nm (12 pmol) operation controlling strands and inputs to form a system with a final volume of 50 µL for the first‐time using. 120 nm (6 pmol) c‐and1, 240 nm (12 pmol) c‐clips/inputs were added for the first time erasing. The concentration of inputs/clips and C‐input/clips would increase 10 nm each round. All experiments depicted in the figure were replicated at least twice, with consistent trends observed.

After verifying the feasibility of basic CLB‐based logic gates, we tried to build a cascaded circuit containing more logical operations to further reveal the power of the CLB design. The cascaded circuit is illustrated in **Figure** **3**a and named as “X‐AND”. In the first layer, we chose to place an AND gate rather than an OR gate. Because the AND logic must guarantee a low leakage when the input is (0,0), (0,1), or (1,0), while the OR gate only faces the leakage problem when the input is (0,0). Therefore, the “X‐AND” gate posed a greater challenge to our leakage‐control strategy. By exploring the construction principle of “X‐AND”, we could improve the anti‐leakage techniques, and apply them to the more complex circuits to strengthen the robustness of the CLB‐based circuits. As shown in Figure 3a, there existed two positions in the “X‐AND” circuit where the operation‐controlling strand could be added, and both of them were located in the second layer of the circuit. By adding different clips, each location was able to field program the following functions: OR, AND, and Translation. Its complete logic operation table is shown in Figure 3b. Take “A AND B AND C” for example. or11 and and21 should be added at positions 1 and 2, respectively. When input‐B and input‐C coexisted, they would form a duplex through a 14‐nt complementary region. The input duplex then invaded gate2:output2, eventually dissociating output2. At the same time, input‐A, using or11 as a toehold, invaded gate1:output1 so that output1 could be dissociated. After that, output1 and output2 would form a duplex just like input‐B and input‐C. Finally, the FAM strand could be displaced off, and the whole circuit executed the function of “A AND B AND C”. As for the erasing process, it could be accomplished by adding C‐clips and C‐inputs to the circuit. Then, the FAM strand, as the invader, disassociated And1:And2 from the BHQ strand. Consequently, output1 and output2 took their 14‐nt single‐strand segment as a toehold to hybridize with gate1 and gate2, respectively, completing the reset of the second layer. Since the entire circuit has been restored to the “initial state”, a new set of clip and input strands could be added to reprogram the circuit.

Figure 3d,e, and Figures S3 and S4 (Supporting Information) showed that all the five logic operations of “X‐AND” listed in the truth table can be successfully realized. In all logic operations, the signal reached to plateau within 90 min, which was slower than that of basic logic gates but still relatively fast as a cascaded circuit. Moreover, in some scenarios, the leakage of the “X‐AND” circuit was even below the detection limit of the instrument, so it presented a straight line with a slope close to 0.

The reprogramming process was similar to that described above. When forward reactions were completed, C‐clips and C‐inputs were added to initiate the erasing reaction, so the fluorescence signal began to decrease. However, as a cascaded circuit, the decline of the fluorescence signal could only indicate that the reporter, not the whole system, had been successfully reset. So, we chose to place the system at 37 °C for 90 min, even though the fluorescence signal had already declined to the baseline level. After that, new clips and inputs could be added to complete the following rounds of field programming. As shown in Figure S5 (Supporting Information), we performed reusing (A AND B AND C AND D) for three rounds. Each round's reaction demonstrated a clear distinction between the leakage and the major signal due to the excellent control of the leakage. Never did a leakage signal exceed 25% of the main signal and some of them were even less than 1%. Based on this, we then tried to verify the circuit's ability of logic‐switching. (A AND B AND C AND D) was operated in the first round; (A AND B AND C) in the second round; and (A AND B) in the third round. As shown in Figure 3f–h, we kept the fluorescence signal amplitude stable with an 80% output yield in the final round. To sum up, we successfully constructed a cascaded CLB‐based field‐programmable “X‐AND” circuit with two operation‐controlling sites. The system was capable of field programming into five different logic operations, and one single circuit could be reused three times. The above data firmly proved that CLB‐based design can be used to build cascaded circuits, which further expands its practicability.

It was worth noting that we adjusted the length of the “incumbent toehold”. As shown in Figure 3c, it referred to the segment whose dissociation was spontaneous rather than caused by being displaced by an invading strand.^[^ ^19^ ^]^ Such a domain acted like a clamp, hindering the gate–gate leakage between output1 and output2. Moreover, it also provided resistance to the forward reaction, reducing the extent of the forward reaction. According to the tradeoff shown in Figure S6 (Supporting Information), we set the length of the incumbent toehold to 5‐nt to guarantee a high signal‐to‐noise ratio. In addition, to further explore the performance of the system under multiple times of reusing, we opted to cyclically switch between “A AND B AND C” and “A AND B” until the system collapsed. As shown in Figure S11 (Supporting Information), the system still managed to distinguish the “ON” signal and the “OFF” signal till the 4th round. In the 5th round, the major signal was very close to the cut‐off line, indicating that the system had reached its limit for reusing or logic switching. We believe this result can serve as a reference. If the circuits involved in logic switching are simpler, the circuit might be able to distinguish between the major signal and leakage over more rounds. Conversely, if the circuits involved in logic switching are more complex, the circuit may collapse sooner.

### 2.4 Construction of CLB‐Based Half‐Adder and Half‐Subtractor

Since the ultimate goal of a DNA logic circuit is to build a biological computer on a submicroscopic scale, it is an inevitable trend to imitate and replace the silicon‐based binary operation circuit. Based on the progress we have made, we further wanted to explore whether CLB‐based circuits were capable of field programming binary operations. We chose half‐adder and half‐subtractor for the exploration because they have been generally simulated by various principles of DNA circuits.^[^ ^32^, ^40^, ^49^, ^50^, ^51^ ^]^

**Figure 4:**
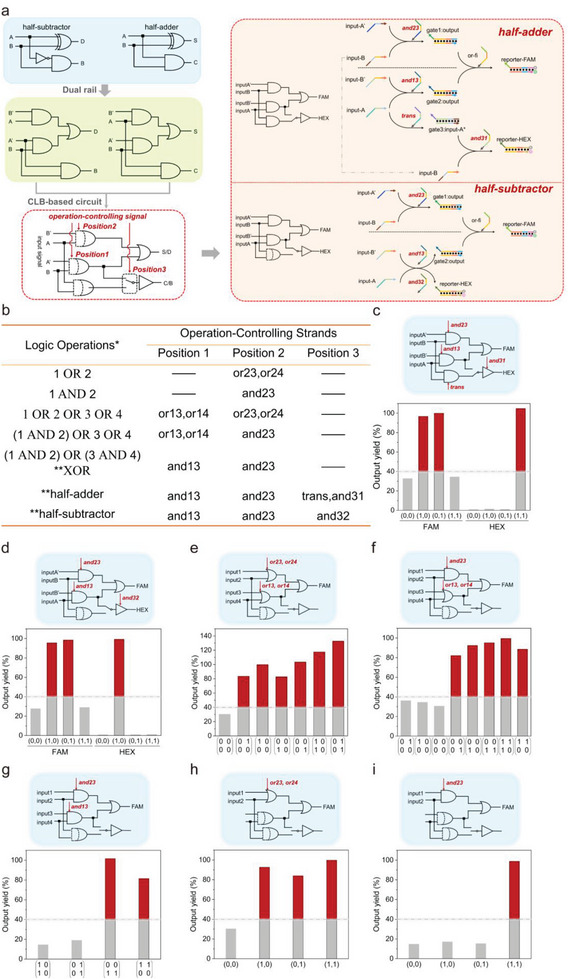
a) Schematic illustration of integrating a half‐adder and a half subtractor into one dual‐rail comprehensive circuit. Inside the dashed box illustrates the digital circuit diagram of half‐adder/subtractor and their corresponding CLB‐based DNA circuit. b) Logic operation table of the integrated comprehensive circuit. ^*^ Input‐1, 2, 3, or 4 in different logic operations does not stand for the same strand.^**^ The corresponding logic operations were dual‐rail. The output yield of half‐adder c), half‐subtractor d), “1 OR 2 OR 3 OR 4” e), “(1 AND 2) OR 3 OR 4” f), XOR g), “1 AND 2” h), “1 OR 2” i). Note that the matrix was arranged in the order of Reactions setup: 100 nm (5 pmol) FAM:BHQ and HEX:HBHQ, 240 nm (12 pmol) gate1:output, gate2:output and gate3:ouputA^*^, 120 nm (6 pmol) or‐fi, 240 nm (12 pmol) operation‐controlling strands and 480‐nm input strands were added sequentially to form a system with final volume of 50 µL. All experiments depicted in the figure were replicated at least twice, with consistent trends observed.

In brief, the most important difference between the previous cascaded circuit and the half‐adder/subtractor was that the latter had two outputs: Sum and Carry for the half‐adder, and Difference and Borrow for the half‐subtractor. Both Sum and Difference = (A XOR B), Carry = (A AND B), Borrow = (A' AND B). The process of integrating the above two logic circuits into one comprehensive CLB‐based circuit is shown in **Figure** **4**a. The circuit was designed to have three operation‐controlling sites. Position 1 and 2 were located in the second layer, where different clips were added to implement Translation/OR/AND, otherwise the corresponding component would be silenced since no clip was added to the site. Position 3 was located at the output end of the Carry/Borrow signal, where different clips decided which upstream components the reporter would connect: the AND gate or the inputs directly. The FAM channel represented SUM/Difference while the HEX channel represented Carry/Borrow. When and13 and and23 were added to Position 1 and 2, respectively, and the HEX reporter was connected to the AND gate by trans and and31, a half‐adder was built. When the connection was switched to the inputs by adding and32, the circuit would become a half‐subtractor. As shown in Figure 4c,d and Figure S7 (Supporting Information), 100‐nm FAM:BHQ and HEX:HBHQ, 240 nm gate1:output, gate2:output and gate3:ouputA^*^, 120‐nm or‐fi, was added first. Then, adding 240 nm corresponding operation‐controlling and 480‐nm input strands enabled the circuit to operate correct logic operations. Both FAM and HEX channel reached a plateau in 90 min with leakage controlled below 35%. It is worth noting that in order to ensure the performance of the half‐adder and half‐subtractor, we have made a series of adjustments. We used a fan‐in strategy (the outputs of two upstream AND gates were used as inputs of the downstream gate) to replace the OR gate at the FAM reporter. This simplification eliminated the need to create another clip and its corresponding output, making the reprogramming reaction much easier. Furthermore, our preliminary findings shown in Figure S8 (Supporting Information) indicated that the 7‐nt incumbent toehold in the HEX reporter, which was consistent with the FAM reporter, provided too much resistance to the invader strand. As a result, there was almost no signal observed in the HEX channel. In order to ensure that the HEX channel is capable of producing a sufficiently high fluorescence signal, it is necessary to shorten the length of the incumbent toehold. However, a 0 nt toehold would not be the best situation because the incumbent toehold will serve as the toehold for c‐input to invade the input:gate duplex during the erasing process. Therefore, the longer the incumbent toehold, the faster and more sufficient the erasing process would be. So, according to Figure S8 (Supporting Information), we believe that a 4 nt incumbent toehold ensures both a higher signal output for the forward reaction and a sufficiently long toehold for the c‐input during the erasing reaction.

We also noted that though the leakage did not hinder the major signal, the half‐adder and half‐subtractor circuits tended to have a greater leakage problem than previous logic circuits. Because both the input of the half‐adder and the half‐subtractor required fan‐out (the output of one upstream gate was used as the input of two downstream gates at the same time), the input concentration needs to be doubled (480 nm). In the dual‐rail half‐adder/ half‐subtractor, inputs first went into the AND gate in the second layer. Consider the case of (0,0), each AND gate had only one input, hence did not output the major signal. However, the higher concentration of input would still push the reaction to proceed, thus increasing the leakage signal. Also, the HEX signal rose more slowly in the half‐adder because of the inherent difference in reaction principles for the half‐adder and half‐subtractor. As shown in Figure 4a, when the system performs half‐adder operations, input A must first enter a Translator (gate3:inputA^*^), thereby producing input A^*^. Subsequently, input A^*^, alongside input B, was able to complete the AND operation, leading to the detachment of the HEX strand. However, when the system executes half‐subtractor operations, input A and input B' can directly invade the HEX reporter in the presence of and32, thereby it does not need the Translator operation compared to half‐adder operations. In summary, the HEX signal of the half‐adder is output by a two‐layer circuit, while the half‐subtractor involves only a single‐layer circuit. Therefore, the rise of the HEX signal in the half‐subtractor is noticeably faster.

In summary, using the CLB‐based design, we managed to integrate the half‐adder and half‐subtractor into one circuit, so researchers only need to add different operation‐controlling strands to obtain corresponding computing elements.

### 2.5 A Comprehensive Erasable Field‐Programmable CLB‐Based DNA Device Enabling 7 Logic Operations Including Half‐Adding and Half‐Subtracting

Furthermore, we tried to expand the function of the circuit by adding different combinations of operation‐controlling strands based on the half‐adder/half‐subtractor circuit, so that the field programmability of this comprehensive circuit could be fully explored. Various combinations of operation‐controlling strands in positions 1–3 and their corresponding logic operations are listed in Figure 4b. Their reaction principles and experimental verifications were illustrated in Figure S9 (Supporting Information) and Figure 4c–i. Besides the half‐adder and half‐subtractor, other logic operations also showed a good signal‐to‐noise ratio and were able to complete the reaction within 90 min.

**Figure 5:**
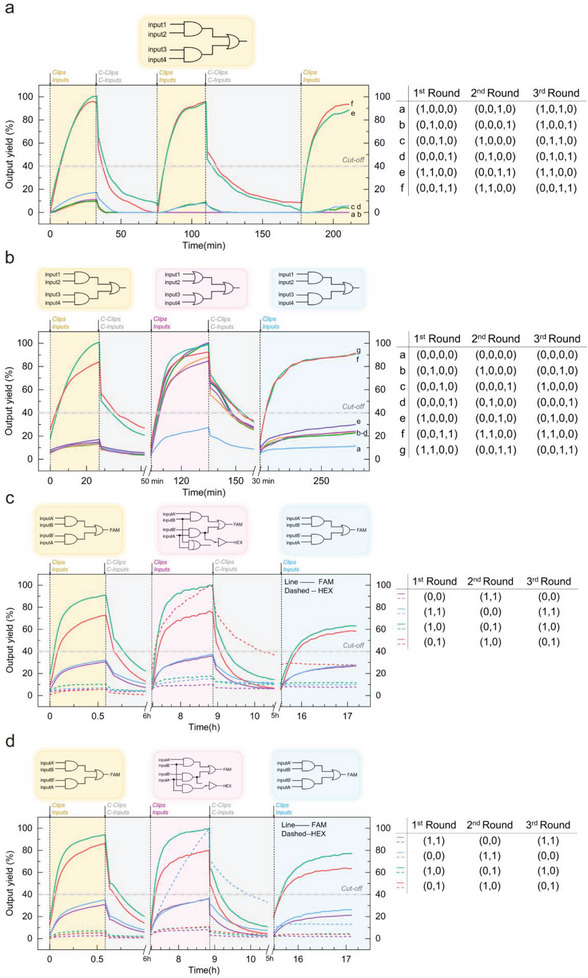
a) Fluorescent curves of XOR gate reusing 3 times. b) Fluorescent curves of the comprehensive logic gate switched in the order of XOR‐(1 OR 2 OR 3 OR 4). c) Fluorescent curves of the comprehensive logic gate switched in the order of XOR‐half adder‐XOR. d) Fluorescent curves of the comprehensive logic gate switched in the order of XOR‐half subtractor‐XOR. Reactions setup: 100 nm (5 pmol) FAM:BHQ and HEX:HBHQ, 240 nm (12 pmol) gate1:output, gate2:output and gate3:ouputA^*^, 120 nm (6 pmol) or‐fi, 240 nm (12 pmol) operation‐controlling strands and 480‐nm input strands were added to form a system with final volume of 50 µL for the first‐time using; 120 nm (6 pmol) c‐or‐fi, 240 nm (12 pmol) C‐clips and 480‐nm C‐inputs were added for the first‐time restoring. The concentration of inputs/clips and C‐input/clips would increase 10 nm each round. All experiments depicted in the figure were replicated at least twice, with consistent trends observed.

After verification of multiple logic operations, we tried to reprogram this circuit. First, we investigated the erasability of the operations that output FAM only. We selected XOR and (1 OR 2 OR 3 OR 4) as representatives, and the results shown in **Figures** **5**a and S10a (Supporting Information) demonstrated that both logic operations could be reused three times. Figure 5b further proved the above 2 logics could be switched to each other back and forth. Based on this, we next tried the logical switching of the half‐adder and the half‐subtractor. As shown in Figure 5c,d and Figure S10b,c (Supporting Information) the circuit could perform three logics in the order of XOR‐half adder/half subtractor‐XOR, or tow logics in the order of half adder/half subtractor‐XOR. In all, we have built a CLB‐based DNA circuit enabling seven logic operations.

It was worth noting that in (1 OR 2 OR 3 OR 4), the output signals of (1,1,0,0) and (0,0,1,1) were significantly higher than 100%. The signal increase might be attributed to the fan‐in strategy, which resulted in 480‐nm output strand invading the reporter in the two preceding cases. This conflicted with the maximum output concentration we had previously explored, but we consider it acceptable because the results were reproducible and did not prevent us from drawing a cutoff line between leakage and the main signal.

Based on the current principle and results, we could summarize the strategy of building circuits using the CLB strategy as follows: First, researchers must use their expertise in digital circuits to design the target circuit with only AND and OR gates. Since we have shown the principles of AND and OR gates, users may then design the appropriate DNA sequence and then perform assembly and cascading according to the digital circuit diagram. In terms of generality, although there may be limitations in constructing logic gates based on our principles in specific scenarios (Discussion S1, Supporting Information), we believe that the aforementioned strategy is sufficient for building the vast majority of logic circuits. As for scalability, our experimental results have demonstrated the feasibility of cascading among Translator, AND, and OR gates. Such cascaded circuits can achieve stable logic switching and reusing. Moreover, based on the dual‐rail logic circuit, combinations of these logic gates are sufficient to realize universal logical operations. Furthermore, the scalability of CLB‐based circuits still holds significant potential for further exploration. In the current system, inputs and operation‐controlling strands are manually added. However, we envision that in the future, by connecting their upstream with DNA aptamer‐based “biological interface” circuits, proteins and other biomacromolecules could participate in the operation and logical switching of CLB circuits.^[^ ^52^, ^53^, ^54^ ^]^ This will significantly enhance the capability of DNA circuits to perform biological signal recognition and computation within the body, bringing them closer to the ultimate goal of a biological computer. Overall, the computing potential and multiple regulatory methods endow the CLB‐based DNA circuit with a wide range of application prospects.

## 3 Conclusion

In summary, inspired by the CLB in digital circuits, we have built the erasable field‐programmable DNA circuit for the first time. Utilizing an allosteric‐clip‐toehold based strand displacement reaction, we managed to design and construct two basic logic gates: AND and OR. Additionally, we used C‐clip and C‐input to complete three consecutive reprogramming of the basic logic gate, which included both erasing and logic‐switching. Based on the above design, we further constructed a cascaded CLB circuit “X‐AND” that could execute five different logic functions and verified the field programmability of the most complex one, “A AND B AND C AND D”. Moreover, using the dual‐rail design, we successfully constructed two basic CLB‐based binary calculators: a half‐adder and a half‐subtractor. Finally, through different combinations of operation‐controlling strands, we could use the comprehensive CLB‐based circuit to realize seven different logic operations and reprogram it according to our needs.

Conclusively, our established CLB‐based DNA circuit could be truly field‐programmed to realize various logic operations. Researchers can obtain the desired circuit only by adding a group of operation‐controlling strands, and its erasability allows us to reuse the same circuit for multiple attempts. We believe that our new circuit design can make DNA circuit programming more efficient, thereby accelerating the birth of a real DNA‐based molecular computer.

## Conflict of Interest

The authors declare no conflict of interest.

## Author Contributions

Y.L. performed the theoretical analysis, designed the experiments, conducted most of the experiments, analyzed the data. Y.Z., H.H., Y.L., H.L., X.L., and J.H. conducted some of the experiments and participated in the discussion of the work. L.W. and H.W. participated in the discussion. L.L., X.Z. and X.X. supervised the whole work, wrote and revised the paper.

## Supporting information

Supporting Information

## Data Availability

The data that support the findings of this study are available from the corresponding author upon reasonable request.
