# Supplementary material for: Erasable and Field Programmable DNA Circuits Based on Configurable Logic Blocks
Source: Adv Sci (Weinh). 2024 May 2;11(26):2400011. doi: 10.1002/advs.202400011 (PMC11234411; doi:10.1002/advs.202400011)
Supplement: Supplementary file 1 — Supporting Information [file ADVS-11-2400011-s001.pdf]

ADVANCED SCIENCE
Open Access

Supporting Information

for *Adv. Sci.*, DOI 10.1002/advs.202400011

Erasable and Field Programmable DNA Circuits Based on Configurable Logic Blocks

Yizhou Liu, Yuxuan Zhai, Hao Hu, Yuheng Liao, Huan Liu, Xiao Liu, Jiachen He, Limei Wang, Hongxun Wang, Longjie Li, Xiaoyu Zhou* and Xianjin Xiao**

Supplementary Information for

Erasable and Field Programmable DNA Circuits Based on Configurable Logic Blocks

Yizhou Liu [a,b], Yuxuan Zhai [a], Hao Hu [b], Yuheng Liao [b], Huan Liu [b], Xiao Liu [b], Jiachen He [b], Limei Wang[a], Hongxun Wang[a], Longjie Li *[,a,b], Xiaoyu Zhou*[,c], Xianjin Xiao*[,b,d]

* To whom correspondence should be addressed.

Correspondence to: Xianjin Xiao, E-mail: xiaoxianjin@hust.edu.cn

Xiaoyu Zhou, E-mail: xzhou74@cityu.edu.hk

Longjie Li, E-mail: lilongjie@whpu.edu.cn

[a] School of Life Science and Technology, Wuhan Polytechnic University, Wuhan 430023, China

[b] Institute of Reproductive Health, Tongji Medical College, Huazhong University of Science and Technology, Wuhan 430030, China

[c] Department of Precision Diagnostic and Therapeutic Technology, City University of Hong Kong Shenzhen Futian Research Institute, Shenzhen 518000, Guangdong, China

[d] Department of Laboratory Medicine, Tongji Hospital, Tongji Medical College, Huazhong University of Science and Technology, Wuhan, 430030, China

Methods

Materials

Oligonucleotides used were purchased from Sangon Biotech (Shanghai). Oligonucleotides were checked by Sangon Biotech by capillary electrophoresis for purity and by electrospray ionization mass spectrometry for identity. All labeled strands (FAM, BHQ, HEX, HBHQ) were high performance liquid chromatography (HPLC) purified by Sangon Biotech. Oligonucleotides were ordered in the dry format and suspended in ethylene diamine tetra acetic acid (Tris-EDTA) buffer. Concentrations were determined by measuring the absorbance at 260 nm using a NanoDrop 2000 spectrophotometer (Thermo Fisher Scientific). Thermopol reaction buffer (20 mM hydrochloric acid (Tris-HCl), 10 mM potassium chloride (KCl), 10 mM $(NH_4)_2SO_4$, 2mM $MgSO_4$ and 0.1% Triton X-100, pH 8.8 at 25°C) were purchased from New England Biolabs.

DNA sequence design

All the DNA strands and DNA complexes were examined by NUPACK server (http://www.nupack.org) and the OligoAnalyzer tool (https://www.idtdna.com/pages/tools/oligoanalyzer) to make sure that there were no significant unwanted secondary structures or unwanted intermolecular interactions. All used sequences are collected in Tables section in supplementary information.

DNA duplex preparation and the setup of the experiment system

The reaction system consists of DNA duplex, single strand DNA, ddH_2O and ThermoPol reaction buffer, and the total volume was 50 μL. If the system is intended for reusing or logic switching, then set the total volume for the first round to be 50 μL.

For detailed manual procedures:

Firstly, strands forming the DNA duplex（gate:output, FAM:BHQ, HEX:HBHQ）were mixed, heated at 85°C for 5min, lowered to 55°C for 10min, and then incubated at 37°C for 30min. Then, clip and input strands were added to the system. Finally, ddH_2O and Thermopol were added to achieve a final volume of 50 μl, with Thermopol constituting

10% of the total volume. The concentration of DNA duplex and single strand DNA was documented in the “reaction setup” part of each caption.

Fluorescence experiments and data analysis

The fluorescence experiments were performed on Q1000 Real-Time PCR System (LongGene, China) at 37°C .The excitation wavelength for 6-FAM was 485 nm, and the emission wavelength was 528 nm. The excitation wavelength for HEX was 535 nm, and the emission wavelength was 555 nm. Data acquired by the OPTIMAL software were exported to a Microsoft Excel file, which was subsequently imported, analyzed, and plotted using ORIGIN software. For each experiment presented in the article, it has been independently replicated at least twice, with consistent trends observed. The mean of multiple replicates was calculated and utilized for generating the curves or bar graphs. For each curve or bar, the fluorescence data were usually normalized using the minimum fluorescence intensity as “0%”. The maximum fluorescence intensity generated by the optimal concentrations of Clip and input will be normalized to 100% (average of the last three data points) as “100%”. The fluorescence signals of the second and third rounds during reuse and logic switching require volume correction to compensate for the fluorescence signal loss caused by system dilution. $S_A = \frac{S_0 \times V_A(\mu L)}{50(\mu L)}$,where S_0 stood for the raw fluorescence signal value, V_A for the volume of the sample at the time of measurement, and S_A for the corrected fluorescence signal.

Erasability and Reusing/ Logic Switching

The preparation of solutions and measurement procedures are the same as described above before the c-clip and c-input strands were added.

For each round’s erasability of each reaction, the complementary strands (c-clips and c-inputs) were added with equal quantity to the operation-controlling strands (clips) and input strands in the forward reaction. Then the fluorescence was measured.

For each round’s reusing/logic switching of each reaction, the operation-controlling strands and input strands were added, with a concentration increase of 10 nM compared to the previous round. Then the fluorescence was measured.

Tables

Table S1. The logic operation table of the digital CLB circuit illustrated in Fig. 1a

C_0	C_1	C_2	C_3	Operation
0	0	0	0	0
0	0	0	1	A AND B’
0	0	1	0	A’ AND B
0	0	1	1	A XOR B
0	1	0	0	A’ AND B’
0	1	0	1	NOT B
0	1	1	0	NOT A
0	1	1	1	A NAND B
1	0	0	0	A AND B
1	0	0	1	A
1	0	1	0	B
1	0	1	1	A OR B
1	1	0	0	A XNOR B
1	1	0	1	A OR B’
1	1	1	0	A’ OR B
1	1	1	1	1

TableS2. The sequences used in building CLB-based basic logic gates and verifying its reprogrammability

Name	Sequence(5'→3')
1-FAM	AAGGGTGTTGGTTATGCTATGGGATATGFAM
2-BHQ	BHQCATATCCCATAGCATAACCAACACCCTTACTCGTCACTTCAT
3-or1	ATGAAGTGACGAGTAAGGGTGCAATCAACATCTCCGAAGTGAG
4-or2	ATGAAGTGACGAGTAAGGGTGCATACAATCTCCACGATGTTGG
5-input-o1	GGAGATGTTGATTGTTGGTTATGCTA
6-input-o2	GTGGAGATTGTATGTTGGTTATGCTA
7-and1	ATGAAGTGACGAGTAAGGGTGCATCTATCTCTGACCGATTATC
8-input-a1	GTCAGAGATAGATGGGAATAGAATAGTG
9-input-a2	CACTATTCTATTCCTTGGTTATGCTA
10-c-clip-1	CTCACTTCGGAGATGTTGATTGCACCCTTACTCGTCACTTCAT
11-c-clip-2	CCAACATCGTGGAGATTGTATGCACCCTTACTCGTCACTTCAT
12-c-input-1	TAGCATAACCAACAATCAACATCTCC
13-c-input-2	TAGCATAACCAACATACAATCTCCAC
14-c-clip-3	GATAATCGGTCAGAGATAGATGCACCCTTACTCGTCACTTCAT
15-c-input-3	CACTATTCTATTCCCATCTATCTCTGAC
16-c-input-4	TAGCATAACCAAGGAATAGAATAGTG

Table S3. The sequences used in building CLB-based “X-AND” gate and verifying its reprogrammability

Name	Sequence(5’→3’)
1-FAM	AAGGGTGTTGGTTATGCTATGGGATATGCTATGGGATATGFAM
2-BHQ	BHQCATATCCCATAGCATAACCAACACCCTTACTCGTCACTTCAT
17-gate1	CACTATTCTATTCCCATCTATCTCTGACCACCATTACATCCA
18-gate2	TAGCATAACCAAGGAATAGAATAGTGTGAGGTAAGTGTTG
19-or11	TGGATGTAATGGTGGTCAGAGTGGAGATAGATTGGAGGTTGAG
20-or12	TGGATGTAATGGTGGTCAGAGGTGTTGTGTATTGGAGTGGGAT
21-and11	TGGATGTAATGGTGGTCAGAGTGGTGATGGATAGGTGAGTTGA
22-or21	CAACACTTACCTCACACTATTCACTCACAACTACCCTCAATCC
23-or22	CAACACTTACCTCACACTATTCAACTCAACTATCCCTACACTC
24-and21	CAACACTTACCTCACACTATTCACAACTACATACCATCAACCT
25-input-A1	CCAATCTATCTCCAATAGATGGGAATAGAAT
26-input-B1	GGTAGTTGTGAGTGCTATTCCTTGGTTATGC
27-input-C1	GGATAGTTGAGTTGCTATTCCTTGGTTATGC
28-input-D1	CCAATACACAACACATAGATGGGAATAGAAT
29-input-A2	CCTATCCATCACCACATTCCTACATTCC
30-input-B2	GGTATGTAGTTGTGTGTTGATGTGATGG
31-input-C2	CCATCACATCAACACTATTCCTTGGTTATGC
32-input-D2	GGAATGTAGGAATGATAGATGGGAATAGAAT
33-c-or11	CTCAACCTCCAATCTATCTCCACTCTGACCACCATTACATCCA
34-c-and11	TCAACTCACCTATCCATCACCACTCTGACCACCATTACATCCA
35-c-or21	GGATTGAGGGTAGTTGTGAGTGAATAGTGTGAGGTAAGTGTTG
36-c-and21	AGGTTGATGGTATGTAGTTGTGAATAGTGTGAGGTAAGTGTTG
37-c-input-A1	ATTCTATTCCCATCTATTGGAGATAGATTGG
38-c-input-B1	GCATAACCAAGGAATAGCACTCACAACTACC
39-c-input-A2	GGAATGTAGGAATGTGGTGATGGATAGG
40-c-input-B2	CCATCACATCAACACACAACTACATACC
41-c-input-C2	GCATAACCAAGGAATAGTGTTGATGTGATGG
42-c-input-D2	ATTCTATTCCCATCTATCATTCCTACATTCC

* The complementary strands of input and operation-controlling strands have the same name as themselves but with the letter “c” at the beginning.

Table S4. The combination of inputs strands when “X-AND” gate performed different logic operations

Logic Operations	Inputs
A AND B	29-input-A1, 30-input-B1
A AND B AND C	25-input-A1, 30-input-B2, 31-input-C2
A AND D AND (B OR C)	29-input-A2, 26-input-B1, 27-input-C1, 32-input-D2
(A OR D) AND (B OR C)	25-input-A1, 26-input-B1, 27-input-C1, 28-input-D1
A AND B AND C AND D	29-input-A2, 30-input-B2, 31-input-C2, 32-input-D2

Table S5. The sequences used in building a CLB-based comprehensive circuit and verifying its reprogrammability

Name	Sequence(5’→3’)
1-FAM	AAGGGTGTTGGTTATGCTATGGGATATGFAM
2-BHQ	BHQCATATCCCATAGCATAACCAACACCCTTACTCGTCACTTCAT
43-clip-fi	ATGAAGTGACGAGTAAGGGTGCATCTATCTCTGACCGATTATC
44-output	GTCAGAGATAGATGTTGGTTATGCTA
45-gate1	TAGCATAACCAACATCTATCTCTGACCAACTCACATACCT
46-gate2	TAGCATAACCAACATCTATCTCTGACCACCTTACCACACC
47-HEX	AAGAGGGATAGATGTTGGTTAGGTATAGFAM
48-HBHQ	BHQCTATACCTAACCAACATCTATCCCTCTTCAACTACACATCAC
49-gate3	GGAGTGTGTGTGTGAGAGTGGGAGGATGTAGAGGTTGAGAG
50-input-A*	ATCCTCCCACTCTCACACACACACTCCAC
51-or13	AGGTATGTGAGTTGGTCAGAGGAGTGAAGTGATTGGAGTATGG
52-or14	AGGTATGTGAGTTGGTCAGAGAGTGAGTGTGAAGTGAGTATGG
53-and12	AGGTATGTGAGTTGGTCAGAGGTGGTATGAATGGTGTAGGTAG
54-or23	GGTGTGGTAAGGTGGTCAGAGAGTTATGAGTAGTGATGGTGAG
55-or24	GGTGTGGTAAGGTGGTCAGAGGAGTAAGGAGTGATATGGTGAG
56-and22	GGTGTGGTAAGGTGGTCAGAGTGTAAGTGAATGGATGAGAAGG
57-trans	CTCTCAACCTCTACATCCTCCTGTAAGTGAATGGACTGTCATC
58-and31	GTGATGTGTAGTTGAAGAGGGGAGAGTGGGAGGATTGGAGTAG
59-and32	GTGATGTGTAGTTGAAGAGGGGTGGTATGAATGGTAGGTGATG
60-input11-B’	GTGTGTGTGAGAGTGAATAGATGTTGGTTA
61-input21-A	TCCATTCACTTACAACACTCTCACACACAC
62-input31-A’	ACCATTCATACCACACACACACACTCCAC
63-input41-B	GTGGAGTGTGTGTGAATAGATGTTGGTTA
64-input12	CACTACTCATAACTATAGATGTTGGTTA
65-input22	ATCACTCCTTACTCATAGATGTTGGTTA
66-input32	CAATCACTTCACTCATAGATGTTGGTTA
67-input42	ACTTCACACTCACTATAGATGTTGGTTA
68-c-or13	CCATACTCCAATCACTTCACTCCTCTGACCAACTCACATACCT
69-c-or14	CCATACTCACTTCACACTCACTCTCTGACCAACTCACATACCT
70-c-and12	CTACCTACACCATCTCTCCATACTCTGACCAACTCACATACCT
71-c-or23	CTCACCATCACTACTCATAACTCTCTGACCACCTTACCACACC
72-c-or24	CTCACCATATCACTCCTTACTCCTCTGACCACCTTACCACACC
73-c-and22	CCTTCTCATCCATTCACTTACACTCTGACCACCTTACCACACC
74-c-trans	GATGACAGTCCATTCACTTACAGGAGGATGTAGAGGTTGAGAG
75-c-and31	CTACTCCAATCCTCCCACTCTCCCCTCTTCAACTACACATCAC
76-c-and32	CATCACCTACCATTCATACCACCCCTCTTCAACTACACATCAC
77-c-input11	TAACCAACATCTATTCACTCTCACACACAC
78-c-input21	GTGTGTGTGAGAGTGTTGTAAGTGAATGGA
79-c-input31	GTGGAGTGTGTGTGTGTGGTATGAATGGT
80-c-input41	TAACCAACATCTATTCACACACACTCCAC

81-c-input12	TAACCAACATCTATAGTTATGAGTAGTG
82-c-input22	TAACCAACATCTATGAGTAAGGAGTGAT
83-c-input32	TAACCAACATCTATGAGTGAAGTGATTG
84-c-input42	TAACCAACATCTATAGTGAGTGTGAAGT

* The complementary strands of input and operation-controlling strands have the same name as themselves but with the letter “c” at the beginning.

Table S6. The combination of inputs strands when the comprehensive circuit performed different logic operations

Logic Operations	Inputs
1 OR 2	64-input12, 65-input22
1 AND 2	60-input11-B’, 61-input21-A
1 OR 2 OR 3 OR4	64-input12, 65-input22, 66-input32, 67-input42
(1 AND 2) OR 3 OR 4	60-input11-B’, 61-input21-A, 66-input32, 67-input42
(1 AND 2) OR (3 AND 4) /XOR	60-input11-B’, 61-input21-A, 62-input31-A’, 63-input41-B
half-adder	60-input11-B’, 61-input21-A, 62-input31-A’, 63-input41-B
half-subtractor	60-input11-B’, 61-input21-A, 62-input31-A’, 63-input41-B

Table S7. The sequence used in building the multilayer Translator system

Name	Sequence(5'→3')
1-FAM	AAGGGTGTTGGTTATGCTATGGGATATGFAM
2-BHQ	BHQCATATCCCATAGCATAACCAACACCCTTACTCGTCACTTCAT
43-clip-fi	ATGAAGTGACGAGTAAGGGTGCATCTATCTCTGACCGATTATC
44-output	GTCAGAGATAGATGTTGGTTATGCTA
85-cp2	GGTGTGGTAAGGTGGTCAGAGAGTTATGAGTAGTGGATGTTGG
86-op3	CACTACTCATAACTATAGATGTTGGTTA
87-gate3	TAACCAACATCTATAGTTATGAGTAGTGTGTAATGGTGAAGA
88-cp3	TCTTCACCATTACACACTACTCACCATTACATCCACTACCCTT
89-op4	TGGATGTAATGGTGCATAACTATAGATG
90-gate4	CATCTATAGTTATGCACCATTACATCCACACCTCTTAACACA
91-cp4	TGTGTTAAGAGGTGTGGATGTTGGGAGTAAGAGTGTGAGAGGT
92-op5	CACTCTTACTCTCAAATGGTGCATAACT
93-gate5	AGTTATGCACCATTTGAGAGTAAGAGTGTGTAGTGTTAGATG
94-cp5	CATCTAACACTACACACTCTTCACCATTCACCACATCACTCCA
95-op6	TGTGGTGAATGGTGACTCTCAAATGGTG
96-cp2b	CCAACATCCACTACTCATAACTCTCTGACCACCTTACCACACC
97-op3b	TAACCAACATCTATAGTTATGAGTAGTG
98-cp3b	AAGGGTAGTGGATGTAATGGTGAGTAGTGTGTAATGGTGAAGA
99 -op4b	CATCTATAGTTATGCACCATTACATCCA
100 -cp4b	ACCTCTCACACTCTTACTCCCAACATCCACACCTCTTAACACA
101-op5b	AGTTATGCACCATTTGAGAGTAAGAGTG
102-cp5b	TGGAGTGATGTGGTGAATGGTGAAGAGTGTGTAGTGTTAGATG
103-op6b	CACCATTTGAGAGTCACCATTCACCACA
104-cp4	TGTGTTAAGAGGTGTGGATGTTGGGAGTAAGAGTGTGAGAGGT
105-op5	CACTCTTACTCTCAAATGGTGCATAACT
106-gate5	AGTTATGCACCATTTGAGAGTAAGAGTGTGTAGTGTTAGATG
107-cp5	CATCTAACACTACACACTCTTCACCATTCACCACATCACTCCA
108-op6	TGTGGTGAATGGTGACTCTCAAATGGTG

Figures

Figure S1. Optimization of CLB-based logic gate reactant concentration

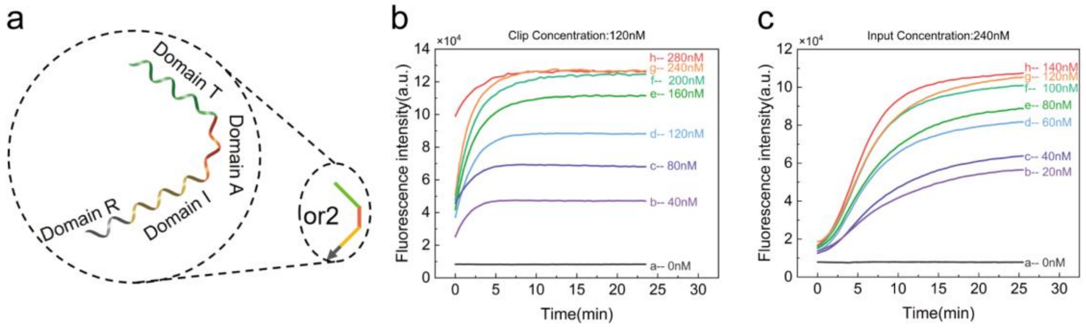

(a) Schematic illustration of the structure of an allosteric clip. (b) Fluorescence curves of allosteric clip-based strand displacement reaction with different concentrations of Input. The concentration of FAM:BHQ were fixed at 100nM and the concentration of Clip were fixed at 120nM. (c) Fluorescence curves of allosteric clip-based strand displacement reaction with different concentrations of Clip. The concentration of FAM:BHQ were fixed at 100nM and the concentration of Input were fixed at 240nM. All experiments depicted in the figure were replicated at least twice, with consistent trends observed.

Figure S2. The comparison between traditional and allosteric clip-mediated strand displacement

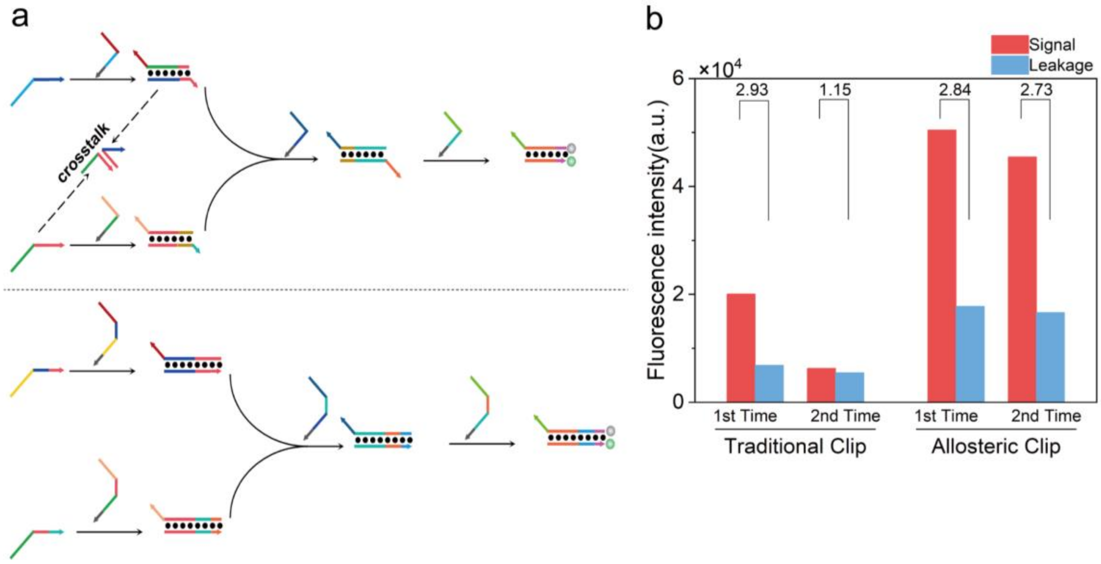

(a) Schematic illustration of a secondary circuit realized by traditional clips (up) and allosteric clips (down) respectively. (b) The comparison of the fluorsecent intensity and signal-to-noise ratio when reusing the above circuits twice. All experiments depicted in the figure were replicated at least twice, with consistent trends observed.

Figure S3. The schematic illustration of various logic operations realized by CLB-based “X-AND” gate

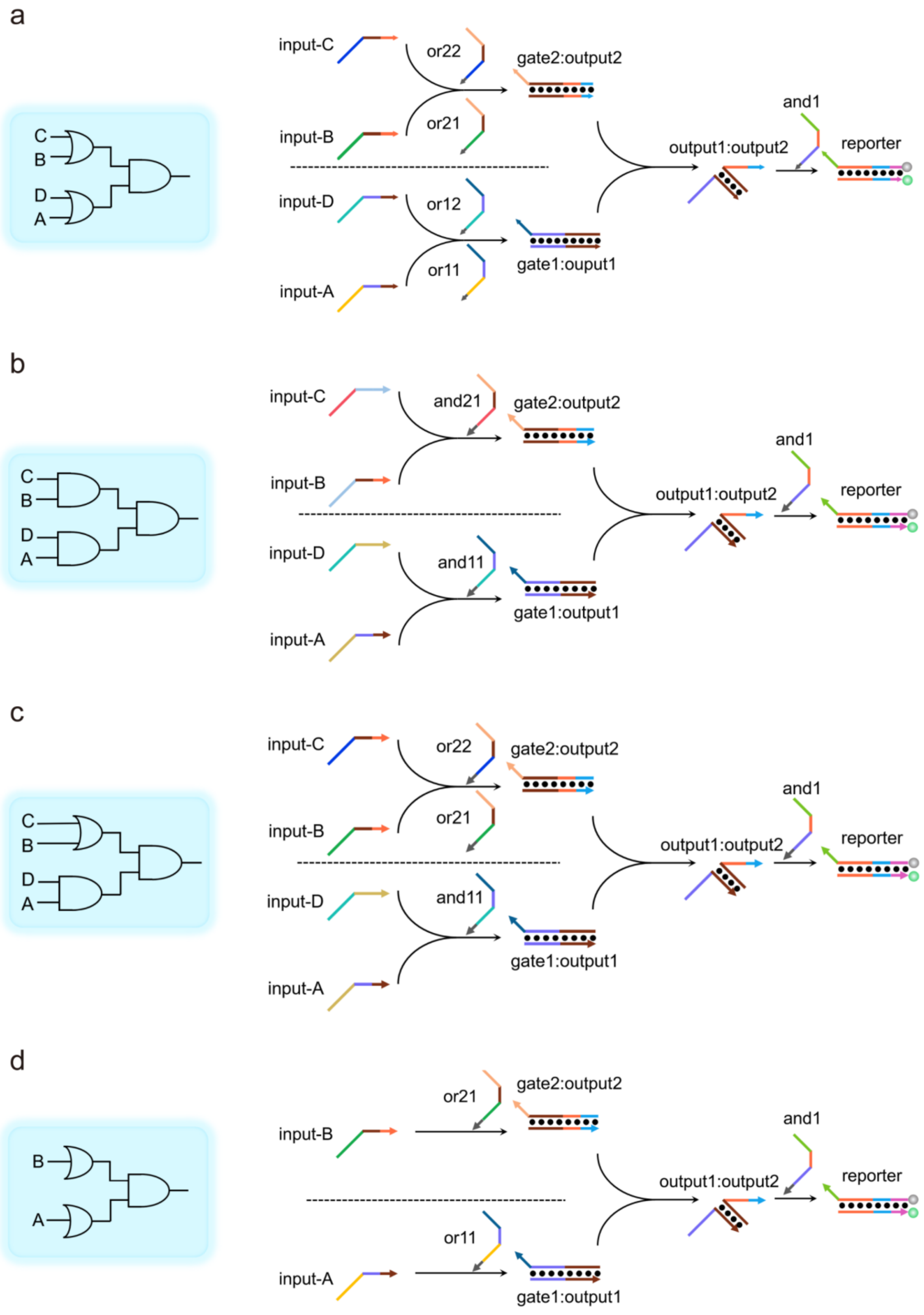

(a-d) The digital circuit diagrams and schematic illustration of corresponding DNA circuits of various logic operations realized by CLB-based “X-AND” gate: “(A OR D) AND (B OR C)” (a), “ A AND B AND C AND D” (b), “A AND D AND (B OR C)” (c), “A AND B”(d).

Figure S4. The experimental verification of various logic operations realized by CLB-based "X-AND" gate

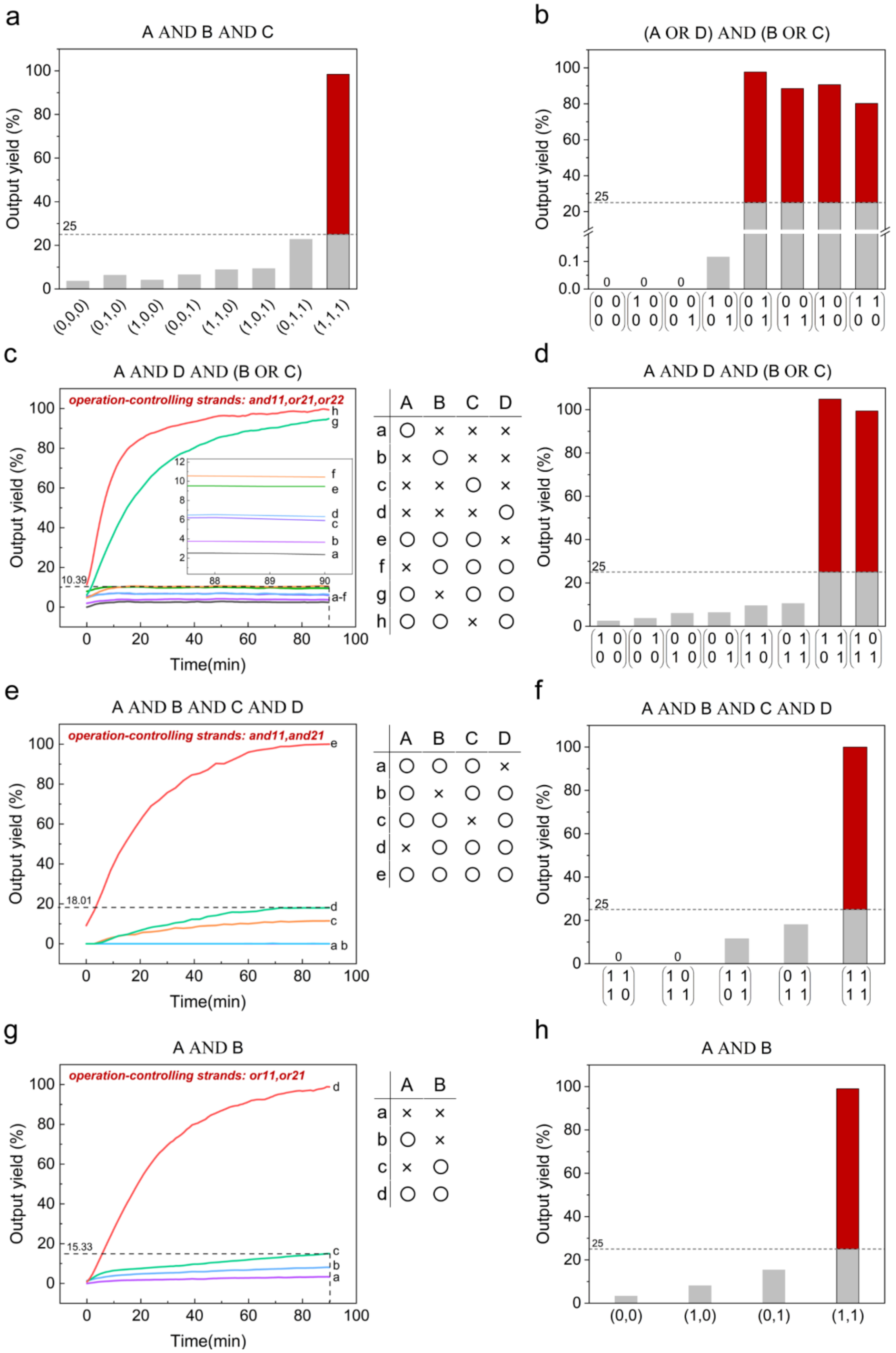

(a-b) The final output yield of “A AND B AND C” (a), “(A OR D) AND (B OR C)” (b). (c-d) The fluorescent curves (c) and the final output yield (d) of “A AND D AND (B OR C)” with different input combinations. (e-f) The fluorescent curves (e) and the final output yield (f) of “A AND B AND C AND D” with different input combinations. (g-h) The fluorescent curves (g) and the final output yield (h) of “A AND B AND C AND D” with different input combinations.Note that the matrix were arranged in the order of $\begin{pmatrix} A & B \\ C & D \end{pmatrix}$ Reactions setup: 100 nM (5 pmol)FAM:BHQ, 240 nM (12 pmol) gate2:output2 and gate1:output1, 120 nM (6pmol) and1, 240 nM (12 pmol) operation controlling strands and inputs were added sequentially to form a system with final volume of 50 μL. All experiments depicted in the figure were replicated at least twice, with consistent trends observed.

Figure S5. The reuse of "X-AND" gate

a

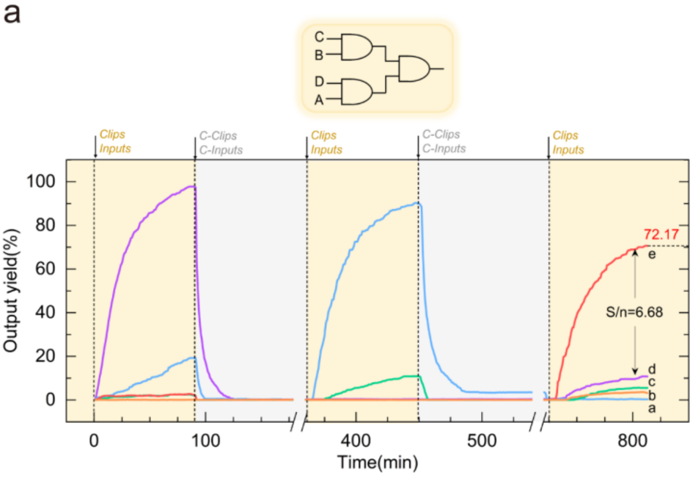

	1st Round	2nd Round	3rd Round
a	(0,1,1,1)	(1,1,1,1)	(1,1,0,1)
b	(1,1,0,1)	(1,0,1,1)	(0,1,1,1)
c	(1,1,1,0)	(0,1,1,1)	(1,1,1,0)
d	(1,1,1,1)	(1,1,0,1)	(1,0,1,1)
e	(1,0,1,1)	(1,1,1,0)	(1,1,1,1)

(a) The fluorescent curves of reusing "A AND B AND C AND D" with different input combinations for 3 times. Reactions setup: 100 nM (5 pmol) FAM:BHQ, 240 nM (12 pmol) gate2:output2 and gate1:output1, 120 nM (6 pmol) and1, 240 nM (12 pmol) operation controlling strands and inputs to form a system with final volume of 50 μL for the first-time using. 120 nM (6 pmol) c-and1, 240 nM (12 pmol) c-clips/inputs were added for the first-time erasing. The concentration of inputs/clips and C-input/clips would increase 10 nM each round. All experiments depicted in the figure were replicated at least twice, with consistent trends observed.

Figure S6. The optimization of the incumbent toehold length of gate:output in CLB-based "X-AND" gate

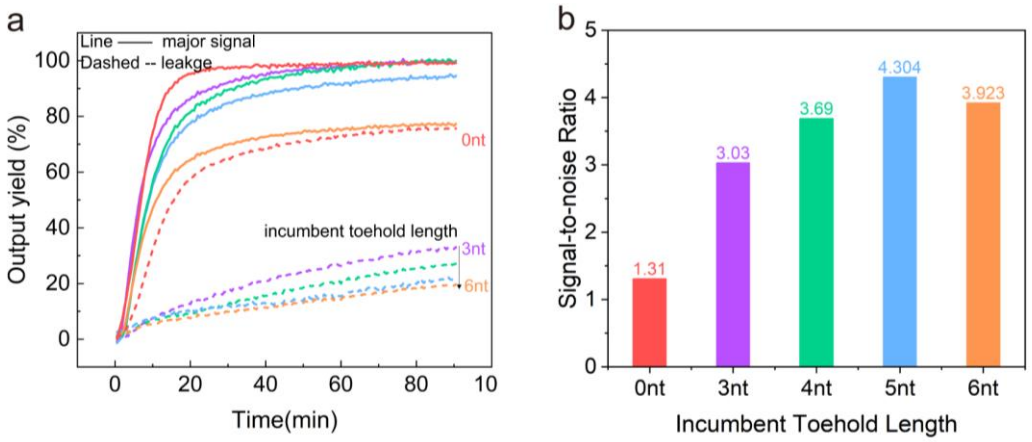

(a-b) The fluorescent curves (a) and Signal-noise-ratio (b) of "A AND B" realized by CLB-based "X-AND" gate with different incumbent toehold length of gate:output. The adjustment of incumbent toehold length was realized by changing the length of input strands. Reactions setup: 100 nM (5 pmol) FAM:BHQ, 240 nM (12 pmol) gate2:output2 and gate1:output1, 120 nM (6 pmol) and1, 240 nM (12 pmol) or11, or21 and inputs were added sequentially to form a system with final volume of 50 µL. Leakage was generated by only adding input B. All experiments depicted in the figure were replicated at least twice, with consistent trends observed.

Figure S7. The experimental verification of CLB-based half-adder and half-subtractor

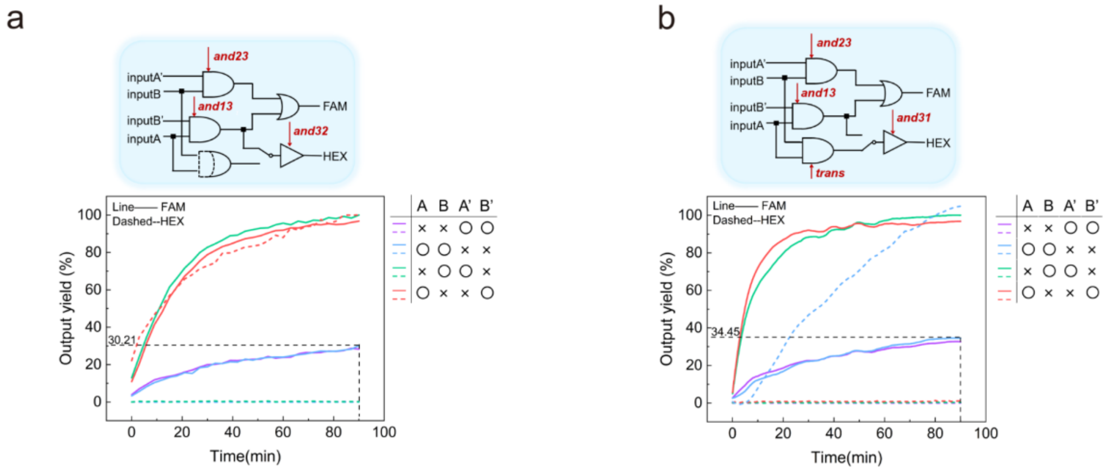

(a-b) The fluorescent curves of CLB-based half-adder (a) and half-subtractor (b). Reactions settup: 100 nM (5 pmol) FAM:BHQ and HEX:HBHQ, 240 nM (12 pmol) gate1:output, gate2:output and gate3:ouputA*, 120 nM (6 pmol) or-fi, 240 nM (12 pmol) operation-controlling strands and 480 nM (24 pmol) input strands were added sequentially to form a system with final volume of 50 µL. All experiments depicted in the figure were replicated at least twice, with consistent trends observed.

Figure S8. The optimization of incumbent toehold length of HEX:HBHQ duplex in the comprehensive CLB-based circuit

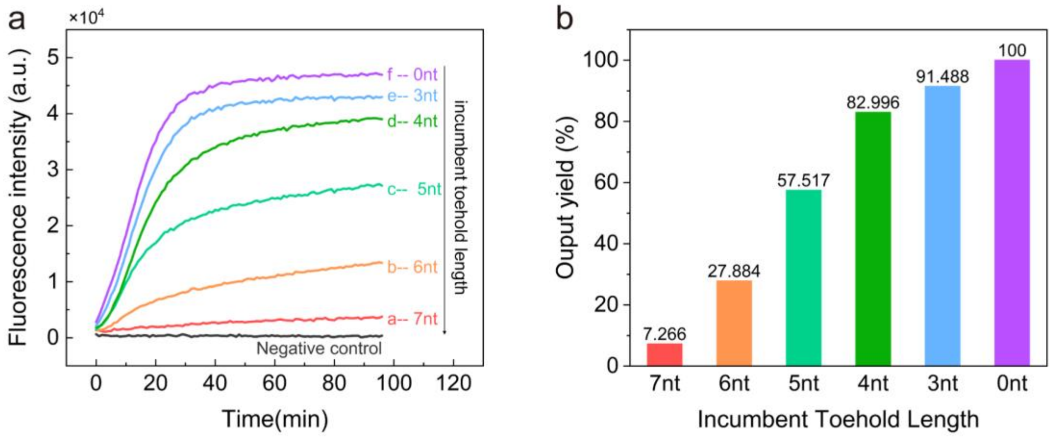

(a-b) The fluorescent curves (a) and the final output yield (b) of input-A:input-B' invading HEX:HBHQ with different incumbent toehold length through and32. The incumbent toehold length was adjusted by changing the length of input strands. Reactions setup: 100 nM (5 pmol) HEX:HBHQ, 120 nM (6 pmol) and32, 240 nM (12 pmol) input-A and input-B' were added sequentially to form a system with final volume of 50 µL. Negative control was generated by adding neither input strand. All experiments depicted in the figure were replicated at least twice, with consistent trends observed.

Figure S9. The schematic illustration and experimental verification of various logic operations realized by CLB-based comprehensive circuit.

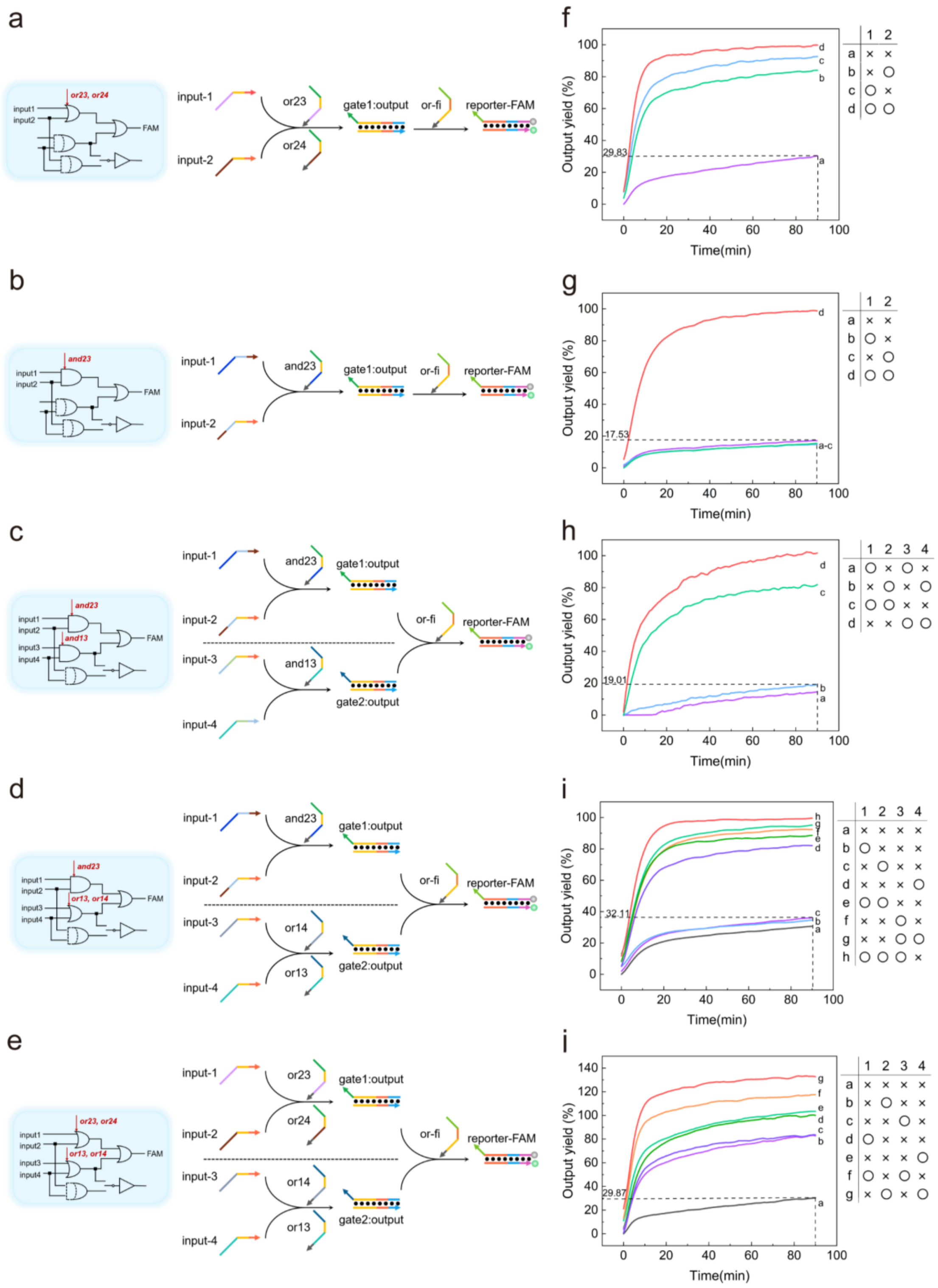

(a-b) The digital circuit diagram and schematic illustration of corresponding DNA circuits of “1 OR 2” (a), “1 AND 2” (b), “(1 AND 2) OR (3 AND 4)” (c), “(1 AND 2) OR 3 OR 4” (d), “1 OR 2 OR 3 OR 4”(e), along with their fluorescent curves(f-j). Reactions setup: 100 nM (5 pmol) FAM:BHQ and HEX:HBHQ, 240 nM (12 pmol) gate1:output, gate2:output and gate3:ouputA*, 120 nM (6 pmol) or-fi, 240 nM (12 pmol) operation-controlling strands and 480 nM (24 pmol) input strands were added sequentially to form a system with final volume of 50 μL. All experiments depicted in the figure were replicated at least twice, with consistent trends observed.

Figure S10. The experimental verification of the reprogrammability of a CLB-based comprehensive circuit

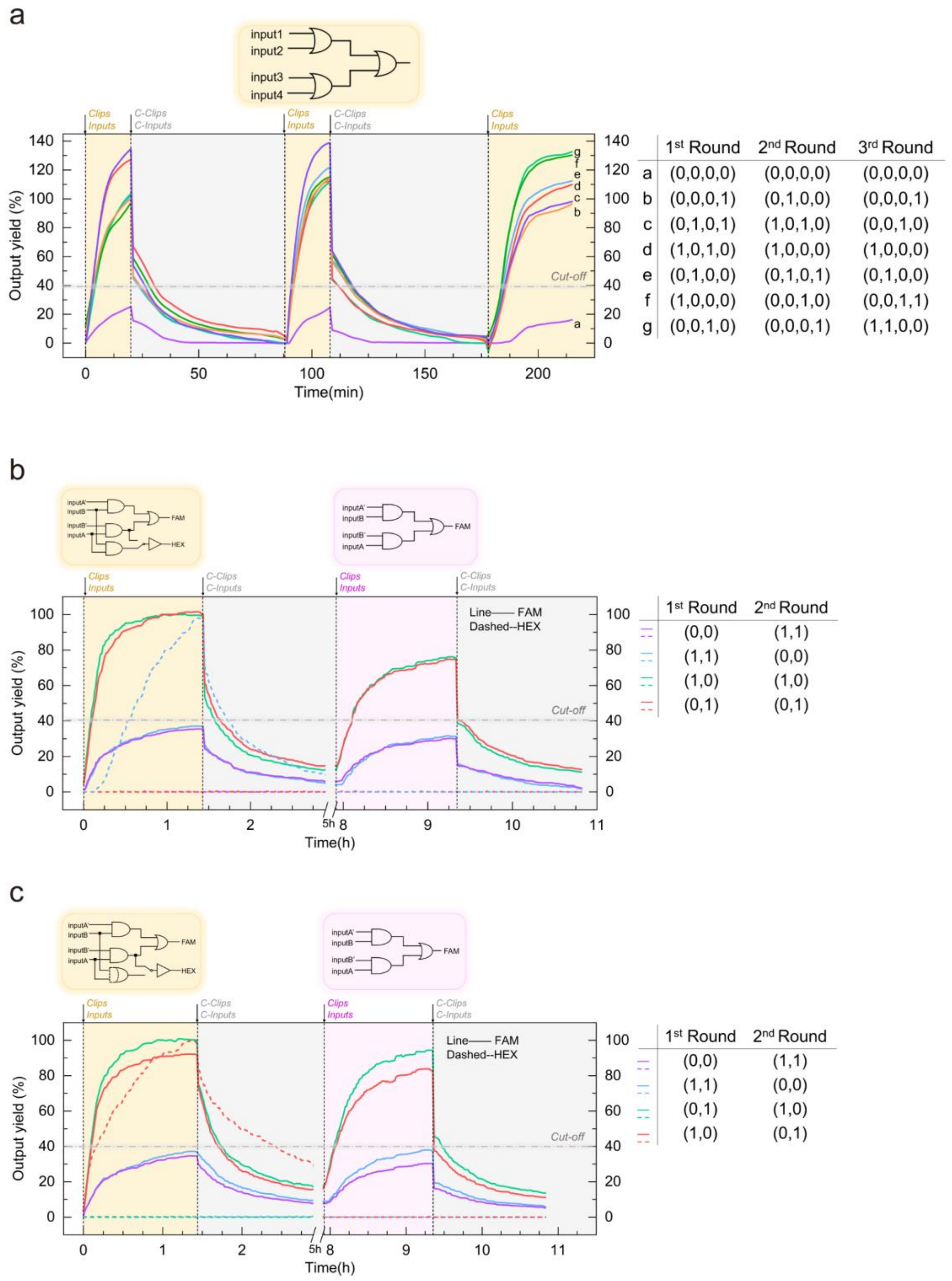

(a) The fluorescent curves of the circuit reusing the logic “1 OR 2 OR 3 OR 4” for 3 times. (b) The fluorescent curves of the circuit opearating half-adder and then reprogrammed to XOR. (c) The fluorescent curves of the circuit opearting hal-subtractor and then reprogrammed to XOR. Reaction settup: 100 nM (5 pmol) FAM:BHQ and HEX:HBHQ, 240 nM (12 pmol) gate1:output, gate2:output and gate3:ouputA*, 120 nM (6 pmol) or-fi, 240 nM (12 pmol) operation-controlling strands and 480 nM (24 pmol) input strands were added to form a system with final volume of 50 μL for the first-time using; 120 nM (6 pmol) c-or-fi, 240 nM (12 pmol) C-clips and 480 nM (24 pmol) C-inputs were added for the first-time restoring. The concentration of inputs/clips and C-input/clips would increase 10 nM each round. All experiments depicted in the figure were replicated at least twice, with consistent trends observed.

Figure S11. Exploration of the performance of the system under multiple times of reusing (more than 3 rounds)

a

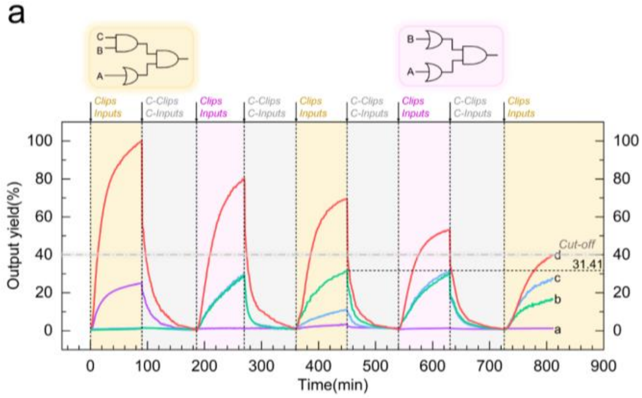

	1st Round	2nd Round	3rd Round	4th Round	5th Round
a	(0,1,1)	(0,0)	(1,1,0)	(0,0)	(1,0,1)
b	(1,0,1)	(0,1)	(0,1,1)	(0,1)	(1,1,0)
c	(1,1,0)	(1,0)	(1,0,1)	(1,0)	(0,1,1)
d	(1,1,1)	(1,1)	(1,1,1)	(1,1)	(1,1,1)

Experiment result of logic switching between “A AND B AND C” and “A AND B” back and forth for 5 rounds till it totally collapse. Reactions setup: 100 nM (5 pmol) FAM:BHQ, 240 nM (12 pmol) gate2:output2 and gate1:output1, 120 nM (6 pmol) and1, 240 nM (12 pmol) operation controlling strands and inputs to form a system with final volume of 50 μL for the first-time using. 120 nM (6 pmol) c-and1, 240 nM (12 pmol) c-clips/inputs were added for the first-time erasing. The concentration of inputs/clips and C-input/clips would increase 10 nM each round. All experiments depicted in the figure were replicated at least twice, with consistent trends observed.

Supplementary discussion 1. The generality of CLB-based DNA circuit.

As for generality, we believe this strategy is adequate for the construction of most digital logic circuits. Since we have shown the principles of AND and OR gates, users may design the appropriate DNA sequence and then perform cascading and assembly according to the digital circuit diagram. However, theoretically, our reaction principle would be limited in the following situations: First, three or more inputs have an AND logic with each other. Take the three-person voting device as an example. As shown in Fig.S12 below, since our proposed AND gate principle required a 14-nt complementary segment between the two inputs, after constructing (A AND B) and (C AND B), it is impossible to build (A AND C). Second, Both AND and OR logic exist between two inputs. Fig.S13 illustrated a dual-rial 4-bit square rooting circuit, in which input a2 and a3 have AND and OR logic simultaneously. As we have mentioned above, the AND logic requires a 14-nt complementary segment however the OR logic requires the 14nt near 3' end to be consistent. Third, when an AND gate have more than two inputs, this multi-input AND gate will have to be split into multiple layers of two-input AND gates. Because our proposed AND gate could only take 2 inputs. Take the A AND B AND C in Fig.3 of the manuscript as an example. And fourth, when an AND logic needs to be built between two inputs which already has an AND logic with other inputs respectively, then a Translator needs to be introduced to convert the DNA sequence, so that the complementary segment of the two rises from 7nt to 14nt. Take the design of half-adder in Fig.4 of the manuscript as an example. Both input-B and input-A have an AND logic with another sequence. In order to build an AND logic between them, input-A has to firstly go into a Translator to convert the sequence. Despite such limitations, we have proved the system's generality by designing the schematic illustration of several complex logic circuits. Such as NAND, 3-input XOR and full-adder (FigS14-S16 below). And it is noteworthy that even within a 4-bit open circuit, researchers can construct the majority of logic gates based on our principles, with the exception of a2 AND a3.

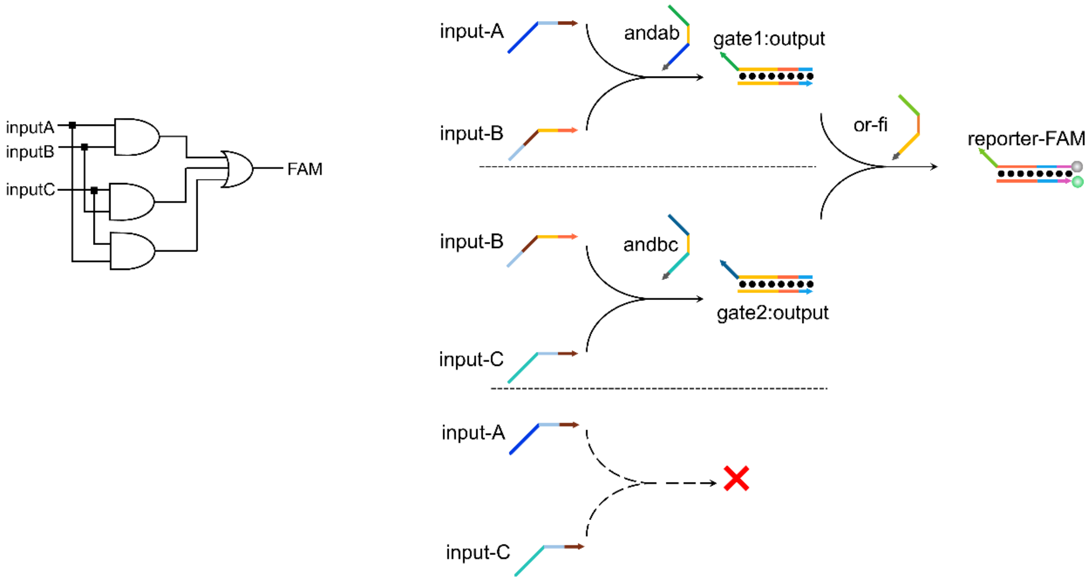

Figure S12. The schematic illustraiton of a three-person voting device.

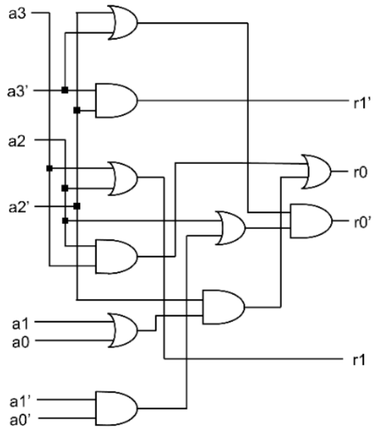

Figure S13. The schematic illustraiton of a dual-rail 4-bit square rooting circuit.

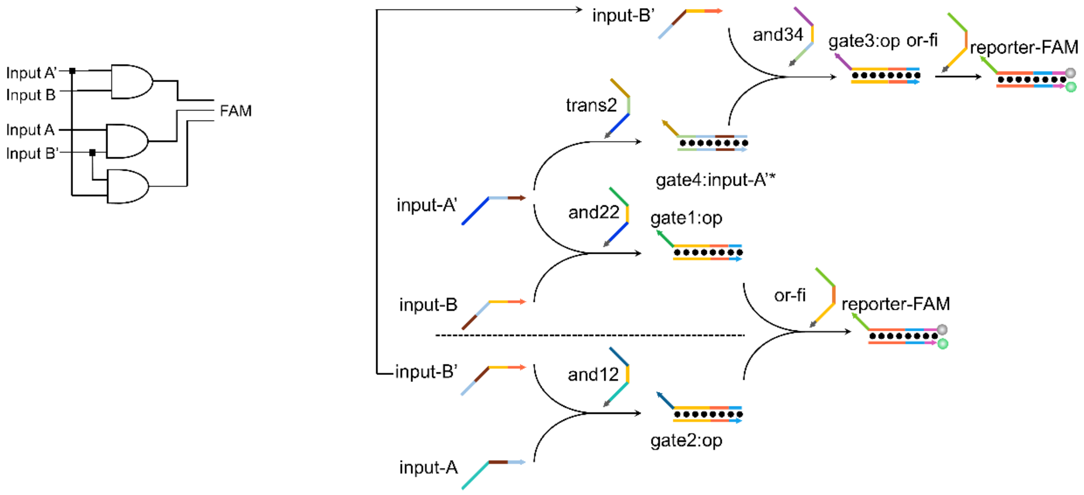

Figure S14 The schematic illustraiton of a NAND gate

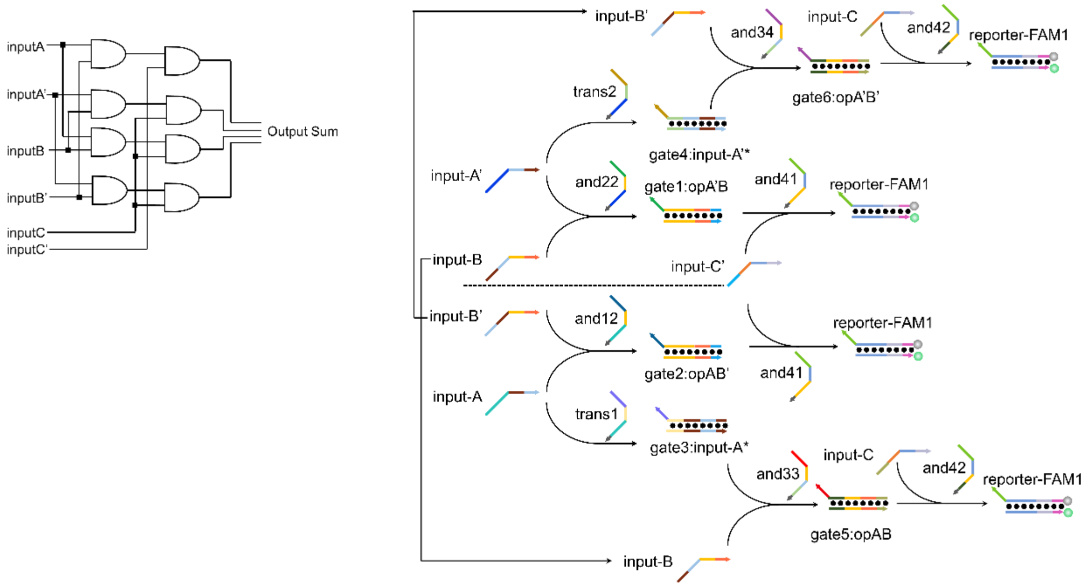

Figure S15 The schematic illustraiton of a 3-input XOR gate.

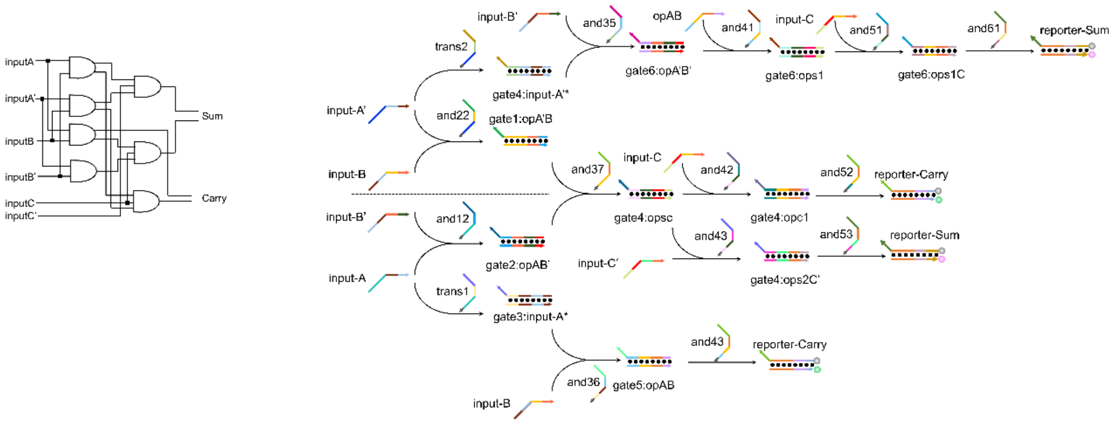

Fig.S16 The schematic illustraiton of a full-adder.

Supplementary discussion 2. The Comparison between DNA logic circuits and traditional silicon-based circuits

Until now the performance of DNA circuits in vitro is still significantly lower than that of traditional silicon-based circuits. However, when considering the operation in the microenvironment, especially in the biological environment, the silicon-based circuit encounters fundamental difficulties. Because of the large size of silicon-based chips (usually measured in cubic millimeters to cubic centimeters), it is unthinkable to use them to build nanodevices that interact directly with biological macromolecules or cells. Moreover, the general silicon-based circuit cannot work properly in the electrolyte, not to mention the complex intracellular microenvironment[1]. The good biocompatibility and compact size of DNA make it a good substitute for silicon-based circuits in the above cases.

1. Speed: At present, the DNA circuit mainly adopts two reaction principles, namely, the toehold or enzyme-mediated strand displacement reaction. The former can complete the construction of the system simply by using the DNA strands, which makes the system cheaper and less dependent on the environment. But the drawback is that, as the reviewer said, the reaction takes dozens of minutes to reach equilibrium. The latter benefits from the high efficiency of enzyme catalysis, and the equilibrium of the reaction can be achieved in 10 minutes. However, because the enzyme has the optimum temperature and pH, the reaction conditions are more stringent, and other enzymes may readily disturb the entire system. We chose the toehold-mediated strand displacement reaction as the basic principle because this system can erase the forward reaction only by adding complementary DNA chains. This is more convenient and cheaper than inactivating the enzyme and then adding the reverse reaction-related enzymes. At the same time, we used the design of the allosteric clip, which increased which greatly increases the speed of reaction. Although the equilibrium time remains in the tens of minutes, it has been greatly reduced when compared to traditional TMSD. We expect that as follow-up research progresses, there will be more refined optimization to overcome the reaction's kinetic issues.
2. Cost: At the moment, the most used DNA circuit detection methods are fluorescence detection and PAGE gel electrophoresis. The former is more commonly used because the reaction of the system can be dynamically monitored by using a real-time PCR instrument to achieve more intuitive and rapid detection. Although the fluorescence modification causes some additional cost, the detection of the whole cascade circuit can be completed only by adding modifications to the double strands of the report system at the end. As a result, the amount of fluorescent group modified chain is extremely minimal each time the system is configured. In our experience, 5OD modified strands (FAM-BHQ) are sufficient to configure about 70 DNA circuits. This equates to a requirement for fluorescently modified DNA costing less than $1.30 per circuit. While the PAGE gel electrophoresis, though less expensive, could only be used after the reaction is complete and unable to monitor the reaction in real time. At the same time, it is counterintuitive to portray the system's reaction through the

location of the strip. Furthermore, if the system has more double-stranded DNA, it may be difficult to differentiate.

3. Scalability: in a short period of time, DNA circuits have to require careful design of oligo DNA sequences to ensure less crosstalk and leakage while increasing the efficiency of the desired reaction. These problems are not encountered in traditional silicon-based circuits. But in the field of DNA circuits, they become the main reasons that restrict the performance of the system. The more complex the circuit, the more difficult it is to design sequences with low crosstalk and leakage. This leads us to implement only the reuse and field programming of half-adder/ half-subtractor for the time being. We foresee that the more general and effective anti-leakage methods in the future will help to improve the scalability of DNA circuits.

4. Biocompatibility: DNA is a naturally biocompatible and water-soluble molecule, and almost all biological functions take place in aqueous electrolyte solutions. So DNA can be applied directly to building artificial logic circuits. At present, several studies have proved that DNA circuits can be successfully constructed on the cell membrane and in cells[2–5]. At the same time, as a natural intracellular substance, DNA can easily interact with other intracellular molecules. Therefore, thanks to the aptamers, a variety of biological macromolecules can be directly used as input signals, thus regulating some biological functions[6–8]. However, silicon-based circuits, as mentioned earlier, will be short-circuited in the electrolyte solution and will not work if no additional treatment is applied. Furthermore, it also lacks an aptamer-like interface to convert biological signals into electrical signals for operation and regulation. Therefore, generally speaking, the biocompatibility of DNA gives DNA circuits a great advantage in running in organisms, which is the main reason why DNA circuits still have development potential, although they obviously lag behind silicon-based circuits in performance.

[1] T. Fu, Y. Lyu, H. Liu, R. Peng, X. Zhang, M. Ye, W. Tan, *Trends Biochem Sci* **2018**, *43*, 547–560.

[2] C. Wu, S. Cansiz, L. Zhang, I. T. Teng, L. Qiu, J. Li, Y. Liu, C. Zhou, R. Hu, T. Zhang, C. Cui, L. Cui, W. Tan, *J Am Chem Soc* **2015**, *137*, 4900–4903.

[3] J. Hemphill, A. Deiters, *J Am Chem Soc* **2013**, *135*, 10512–10518.

[4] M. You, Y. Lyu, D. Han, L. Qiu, Q. Liu, T. Chen, C. Sam Wu, L. Peng, L. Zhang, G. Bao, W. Tan, *Nat Nanotechnol* **2017**, *12*, 453–459.

[5] M. You, L. Peng, N. Shao, L. Zhang, L. Qiu, C. Cui, W. Tan, *J Am Chem Soc* **2014**, *136*, 1256–1259.

[6] A. Bertucci, A. Porchetta, E. Del Grosso, A. Patiço, A. Idili, F. Ricci, **n.d.**, DOI 10.26434/chemrxiv.12424658.v1.

[7] S. Ranallo, D. Sorrentino, E. Delibato, G. Ercolani, K. W. Plaxco, F. Ricci, **2022**, DOI 10.33774/chemrxiv-2021-zz8q0.

[8] Q. L. Zhang, L. L. Wang, Y. Liu, J. Lin, L. Xu, *Nat Commun* **2021**, *12*, DOI 10.1038/s41467-021-24962-4.
